# Fatty acid amide hydrolase and 9-lipoxygenase modulate cotton seedling growth by ethanolamide oxylipin levels

**DOI:** 10.1093/plphys/kiac556

**Published:** 2022-12-06

**Authors:** Omar Arias-Gaguancela, Mina Aziz, Kent D Chapman

**Affiliations:** BioDiscovery Institute, Department of Biological Sciences, University of North Texas, Denton, Texas 76203, USA; BioDiscovery Institute, Department of Biological Sciences, University of North Texas, Denton, Texas 76203, USA; BioDiscovery Institute, Department of Biological Sciences, University of North Texas, Denton, Texas 76203, USA

## Abstract

Polyunsaturated *N*-acylethanolamines (NAEs) can be hydrolyzed by fatty acid amide hydrolase (FAAH) or oxidized by lipoxygenase (LOX). In Arabidopsis (*Arabidopsis thaliana*), the 9-LOX product of linoleoylethanolamide, namely, 9-hydroxy linoleoylethanolamide (9-NAE-HOD), is reported to negatively regulate seedling development during secondary dormancy. In upland cotton (*Gossypium hirsutum* L.), six putative *FAAH* genes (from two diverged groups) and six potential *9-LOX* genes are present; however, their involvement in 9-NAE-HOD metabolism and its regulation of seedling development remain unexplored. Here, we report that in cotton plants, two specific FAAH isoforms (GhFAAH Ib and GhFAAH IIb) are needed for hydrolysis of certain endogenous NAEs. Virus-induced gene silencing (VIGS) of either or both *FAAHs* led to reduced seedling growth and this coincided with reduced amidohydrolase activities and elevated quantities of endogenous 9-NAE-HOD. Transcripts of *GhLOX21* were consistently elevated in *FAAH*-silenced tissues, and co-silencing of *GhLOX21* and *GhFAAH* (*Ib* and/or *IIb*) led to reversal of seedling growth to normal levels (comparable with no silencing). This was concomitant with reductions in the levels of 9-NAE-HOD, but not of 13-NAE-HOD. Pharmacological experiments corroborated the genetic and biochemical evidence, demonstrating that direct application of 9-NAE-HOD, but not 13-NAE-HOD or their corresponding free fatty acid oxylipins, inhibited the growth of cotton seedlings. Additionally, VIGS of *GhLOX21* in cotton lines overexpressing *AtFAAH* exhibited enhanced growth and no detectable 9-NAE-HOD. Altogether, we conclude that the growth of cotton seedlings involves fine-tuning of 9-NAE-HOD levels via FAAH-mediated hydrolysis and LOX-mediated production, expanding the mechanistic understanding of plant growth modulation by NAE oxylipins to a perennial crop species.

## Teaser

Ethanolamide oxylipin content is modulated by the cooperation of two amidases and one lipoxygenase to modulate cotton seedling growth.

## Introduction

*N*-Acylethanolamines (NAEs) are fatty acid derivatives that are represented across diverse eukaryotic organisms. In mammals, the NAE anandamide (NAE20:4) is part of the endocannabinoid signaling pathway, and it participates in multiple neurological and behavioral processes (Bruijnzeel et al., 2016; Moreira-Silva et al., 2018; Kilaru & Chapman, 2020). In plants, NAE profiles differ among plant species and tissues analyzed (Chapman et al., 1999; Venables et al., 2005; Blancaflor et al., 2014). NAEs are generally present in highest amounts in seeds, whereas in vegetative tissues, NAE content is lower, as reported for Arabidopsis (*Arabidopsis thaliana*) (Wang et al., 2006b). Generally, polyunsaturated NAEs are present in higher quantities compared with saturated NAEs, with NAE18:2 most often the most abundant NAE type (Wang et al., 2006b; Blancaflor et al., 2014).

Fatty acid made hydrolase (FAAH) is a membrane protein with conserved amidase and esterase activities (McKinney & Cravatt, 2003; Haq & Kilaru, 2020). It was initially identified in mammalian systems (e.g. rat FAAH), where it was found to inactivate endocannabinoid signaling by hydrolysis of the NAEs into free fatty acids (FFA) and ethanolamine (Bracey et al., 2002; Strittmatter et al., 2012). A plant homolog of the rat FAAH was identified first in Arabidopsis (AtFAAH) (Shrestha et al., 2003; Kim et al., 2009). Like the mammalian FAAH, recombinant AtFAAH hydrolyzed a wide range of NAEs into corresponding products in vitro. Similarly, the legume (*Medicago truncatula*) and rice (*Oryza sativa*) FAAHs also were shown to have amidohydrolase activity towards NAEs (Shrestha et al., 2006). Further, AtFAAH was shown to act on *N*-acyl-L-homoserine lactones (Palmer et al., 2014) and NAE oxylipin substrates (Aziz et al., 2019) in vitro. The action of FAAH in plants against a range of bioactive lipid metabolites suggests that the signaling termination via FAAH might be a broadly distributed mechanism in angiosperms.

Analysis of the AtFAAH 3D structure (Aziz et al., 2019) and of FAAH amino acid sequences of multiple angiosperm species revealed that FAAH is phylogenetically distributed in two major groups with different predicted substrate binding cavities (Aziz & Chapman, 2020). Both groups of FAAHs exhibit conserved catalytic residues, but they differ in key residues of the substrate binding pocket that alter its shape and physical properties (Aziz & Chapman, 2020). There is the possibility that these FAAHs may have overlapping functionality towards certain NAEs, while being distinct towards other lyophilic substrates. In upland cotton (*Gossypium hirsutum* L.), six *GhFAAHs* were reported previously (Arias-Gaguancela et al., 2022). *FAAH* group *I* is composed of *GhFAAH Ia*, *Ib*, *Ic*, and these clustered with *AtFAAH*, whereas *GhFAAH IIa*, *IIb*, and *IIc* are part of *FAAH* group *II*. Group I had higher levels of expression in cotton tissues compared with group II, but all *FAAHs* were expressed in multiple tissues with somewhat greater expression in seedlings and mature leaves (Arias-Gaguancela et al., 2022).

In Arabidopsis, *faah-*knockouts and overexpressing (*AtFAAH*) lines made it possible to uncover certain role (s) of NAEs *in planta* (Wang et al., 2006b; Teaster et al., 2007; Cotter et al., 2011). *faah-*mutants did not have dramatic changes to their normal physiology; however, they were hypersensitive to exogenous applications of NAEs with seedlings showing marked reductions in growth and development. Conversely, *AtFAAH* overexpressing seedlings exhibited enhanced growth, early flowering, and exhibited tolerance to a variety of NAEs (e.g. NAE12:0) (Wang et al., 2006b; Teaster et al., 2007, 2012). These findings suggest that the elevated amount of *AtFAAH* in Arabidopsis transgenics promoted a rapid degradation of NAEs, and this was accompanied by enhanced tolerance to inhibitory growth effects of NAEs. Similarly, transgenic cotton seedlings with ectopic expression of *AtFAAH* exhibited tolerance to exogenously applied NAEs (Arias-Gaguancela et al., 2022).

The polyunsaturated NAE, NAE18:2 also can be metabolized by 9-LOX or 13-LOX enzymes to form their respective hydroperoxides and other NAE-oxylipins (Kilaru et al., 2011; Keereetaweep et al., 2015). In Arabidopsis, the 9-LOX product of NAE18:2 (9-NAE-HOD) along with the phytohormone ABA was shown to be involved in seedling growth arrest associated with the process of secondary dormancy (Keereetaweep et al., 2015). The connection between FAAH and LOX metabolism, at least in terms of their ability to metabolize NAEs, was assessed with genetic and pharmacological experiments (Kilaru et al., 2011; Keereetaweep et al., 2013, 2015; Keereetaweep & Chapman, 2016). Arabidopsis *faah*-knockouts supplemented with NAE18:2 had greater levels of 9-NAE-HOD or 13-NAE-HOD compared with wild-type controls (Keereetaweep et al., 2015). By contrast, *AtFAAH* overexpressing lines had barely detectable levels of 9-NAE-HOD or 13-NAE-HOD compared with controls (Keereetaweep et al., 2015). In pharmacological experiments, 9-NAE-HOD was a potent inhibitor of seedling growth and development. Arabidopsis *AtFAAH* overexpressing lines (and ABA signaling mutants) were more tolerant to exogenous 9-NAE-HOD, when compared with wild-type or 9-LOX-impaired mutants. By contrast, exogenously applied 13-NAE-HOD had no overt growth effects in any genotype (Keereetaweep et al., 2015). Hence, in Arabidopsis, FAAH and LOX enzymes competed for polyunsaturated NAEs to modulate the levels of NAE oxylipins, and the resulting levels of 9-NAE-HOD arrested Arabidopsis seedling development in an ABA-dependent manner.

Interestingly, outside of Arabidopsis (and other Brassicaceae), other angiosperms appear to have two groups of FAAHs in their genomes, and many additional LOX isoforms. Hence it is unclear if, or how, the interplay between FAAH and LOX enzymes might influence seedling development in more complex systems. For example, cotton has a total of six FAAH genes (three group I and three group II) (Arias-Gaguancela et al., 2022) and 21 LOX genes (six putative 9-LOXes and 15 putative13-LOXes, grouped in two sub families; type I and II) (Shaban et al., 2018). Here, we utilized virus-induced gene silencing (VIGS) to suppress multiple cotton FAAH and/or 9-LOX isoforms to assess their impact in seedling growth and NAE oxylipin metabolism. Four major findings can be summarized: (1) VIGS of individual FAAHs demonstrated that out of six FAAHs, only two, namely GhFAAH Ib and/or IIb, were associated with reduced seedling growth and elevated endogenous NAE-oxylipin content. (2) Inspection of multiple 9-LOX isoforms revealed that only suppression of *GhLOX21* could restore the reduced seedling growth found in *FAAH*-silenced seedlings, and this coincided with lower 9-NAE-HOD (but not 13-NAE-HOD) levels compared with *FAAH* silenced plants alone. (3) Silencing of the *GhLOX21* in transgenic cotton seedlings overexpressing *AtFAAH*, resulted in enhanced seedling growth, and this coincided with undetectable levels of 9-NAE-HOD. (4) Pharmacological experiments demonstrated that 9-NAE-HOD rather than 13-NAE-HOD or their respective FFA-oxylipins could inhibit seedling growth and development. Collectively, our data indicate that cotton seedling growth is influenced by the adjustment of 9-NAE-HOD levels via FAAH and LOX enzymes, specifically involving the two FAAH genes, *GhFAAH Ib* and *GhFAAH IIb* and the 9-LOX gene *GhLOX21*.

## Results

### *FAAH* silencing impacts cotton seedling growth and NAE profiles

**Figure 1:**
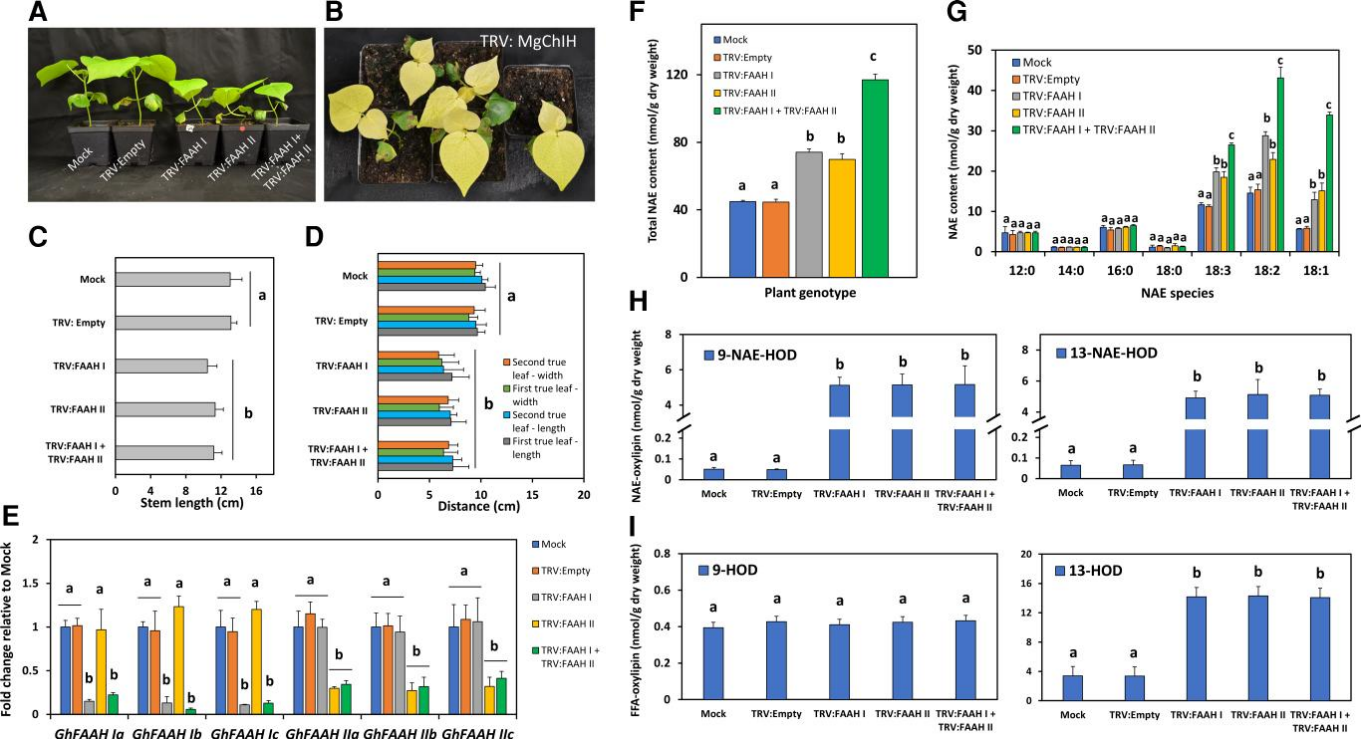
Suppression of *FAAH I* and/or *II* in cotton seedlings. A, Representative images of cotton seedlings inoculated with TRV: FAAH (I and/or II) at 20 days post infiltration (dpi). Mock (infiltration media) and TRV: Empty (vector) were used as negative controls. B, Seedlings infiltrated with TRV: MgChIH to silence *Magnesium chelatase subunit H* were used as positive controls to visualize the timing and uniformity of viral suppression of gene expression. C, Stem length (*n* = 13) and D, leaf width and length (*n* = 13) measurements of *FAAH* silenced plants. Error bars represent the standard deviation (SD). Different letters denote significant differences (*P* < 0.05) by ANOVA with Tukey's post-hoc test. E, RT-qPCR to quantify VIGS silencing efficiency. *UBQ1* was used as the housekeeping gene of normalization. Calculations were made with the delta-delta-Ct (ddCT) method. Error bars represent the SD. Different letters denote significant differences (*P* < 0.05; *n* = 3) by ANOVA with Tukey's post-hoc test. F, Total (unsubstituted/non-oxygenated) NAE content (summed from individual types in G) in FAAH-silenced tissues. Error bars represent the SD. G, Profile of individual NAE types in *FAAH* silenced tissues. H, NAE- and I, FFA-oxylipin profiles. Error bars represent the SD. Different letters denote significant differences (*P* < 0.05; *n* = 3) by ANOVA with Tukey's post-hoc test.

Recently, six FAAH homologs to the Arabidopsis FAAH (AtFAAH) gene were identified and clustered into two groups; FAAH I (GhFAAH Ia, Ib, and Ic) and FAAH II (GhFAAH IIa, IIb, and IIc) (Arias-Gaguancela et al., 2022). However, their physiological role was not explored further. Here, a genetics approach using VIGS helped to assess the requirement of FAAH I and II genes in cotton seedling growth. Initially, two constructs, namely, TRV: FAAH I and TRV: FAAH II, were used to suppress the expression of either all three FAAH I and/or all three FAAH II genes combined (Figure 1). In these experiments, two negative controls (Mock or TRV: Empty) and one positive control (TRV: MgChIH) helped to establish the framework of the silencing system. At 20 dpi, silencing of *FAAH I*, *II* or both groups resulted in overall growth reduction compared with the negative controls (Figure 1A). The albino phenotype observed in the positive control indicated the homogeneity of the VIGS silencing in cotton seedling tissues over this time period (Figure 1B). The stem length, leaf width and length of the primary and secondary leaves were used as quantitative parameters of seedling growth. The average stem length of TRV: FAAH I and/or FAAH II inoculated seedlings was significantly shorter compared with controls (Figure 1C; *P* < 0.05, *n* = 13). In addition, leaf width and length were also reduced compared with mock or empty-vector controls (Figure 1D; *P* < 0.05, *n* = 13). These data suggested that both groups of FAAHs likely participate in cotton seedling growth and development. RT-qPCR was used to quantify the amount of *FAAH* transcript in VIGS-treated seedlings (Figure 1E). Approximately 90% of *FAAH I* transcripts were silenced in TRV: FAAH I inoculated seedlings, whereas 70% of *FAAH II* transcripts were reduced in TRV: FAAH II tissues, and between 80%–90% of *FAAH I* transcripts and 60%–70% of *FAAH II* transcripts were reduced in seedlings where both *FAAH* silencing constructs were co-delivered (Figure 1E; *P* < 0.05, *n* = 3). Notably, multiple comparisons of *FAAH* transcripts in seedlings inoculated with one or two TRV: FAAH silencing constructs revealed no significant differences among themselves (Figure 1E; *P* > 0.05, *n* = 3). These data suggest that silencing of one or two *FAAH* groups can be accomplished with similar efficiency. We evaluated the content and composition of NAE species in *FAAH* silenced tissues. NAE profiles showed that compared with mock or empty-vector controls, total unsubstituted NAE content was the highest in tissues where both TRV: FAAH I and FAAH II were co-delivered (≈62% increase), while seedlings inoculated with either TRV: FAAH I or FAAH II increased ≈40% and 36%, respectively (Figure 1F; *P* < 0.05, *n* = 3). Inspection of individual NAE species revealed no significant changes in amounts of saturated NAEs (NAE12:0, NAE14:0, NAE16:0, and NAE18:0) in either one of the FAAH silenced treatments, whereas unsaturated NAEs (NAE18:3, NAE18:2, and NAE18:1) were significantly elevated in all *FAAH-*silenced plants (Figure 1G; *P* < 0.05, *n* = 3). NAE18:2 accumulated the most in either group of the *FAAH*-silenced seedlings, and also was the highest increase measured in tissues where all *FAAHs* were silenced. Similar results were noted for NAE18:3 and NAE18:1 as well. Taken together, these data revealed that two groups of FAAHs can cooperate to regulate NAE levels, especially the unsaturated 18C NAE species.

Given the importance of LOX-derived NAEs in Arabidopsis growth modulation (Kilaru et al., 2011; Keereetaweep et al., 2013, 2015), we also tested whether endogenous NAE-hydroxides or their corresponding FFA were affected by *FAAH* suppression (Figure 1, H, and I). Hydroperoxides here were fully reduced to hydroxides for increased stability during quantification. Indeed, 9-NAE-HOD levels in TRV: FAAH I and/or FAAH II inoculated seedlings were dramatically elevated- ≈100-fold higher than mock or empty-vector controls (Figure 1H; left panel, *P* < 0.05, *n* = 3). 13-NAE-HOD also exhibited a similar marked accumulation (Figure 1H; right panel, *P* < 0.05, *n* = 3). The FFA oxylipin, 9-HOD, remained unchanged in all treatments (Figure 1I; left panel, *P* > 0.05, *n* = 3), whereas the content of 13-HOD in both of *FAAH* silenced treatments was ≈four-fold higher than without *FAAH* suppression (Figure 1I; right panel, *P* < 0.05, *n* = 3). These data indicated that ethanolamide oxylipins derived from NAE 18:2, namely 9-NAE-HOD and 13-NAE-HOD, markedly accumulated in tissues to a similar extent where either or both sets of *FAAH* genes were silenced, and to a much more dramatic degree than the precursor NAE 18:2 itself.

### A 9-LOX cooperates in FAAH-mediated regulation of NAE-oxylipins

**Figure 2:**
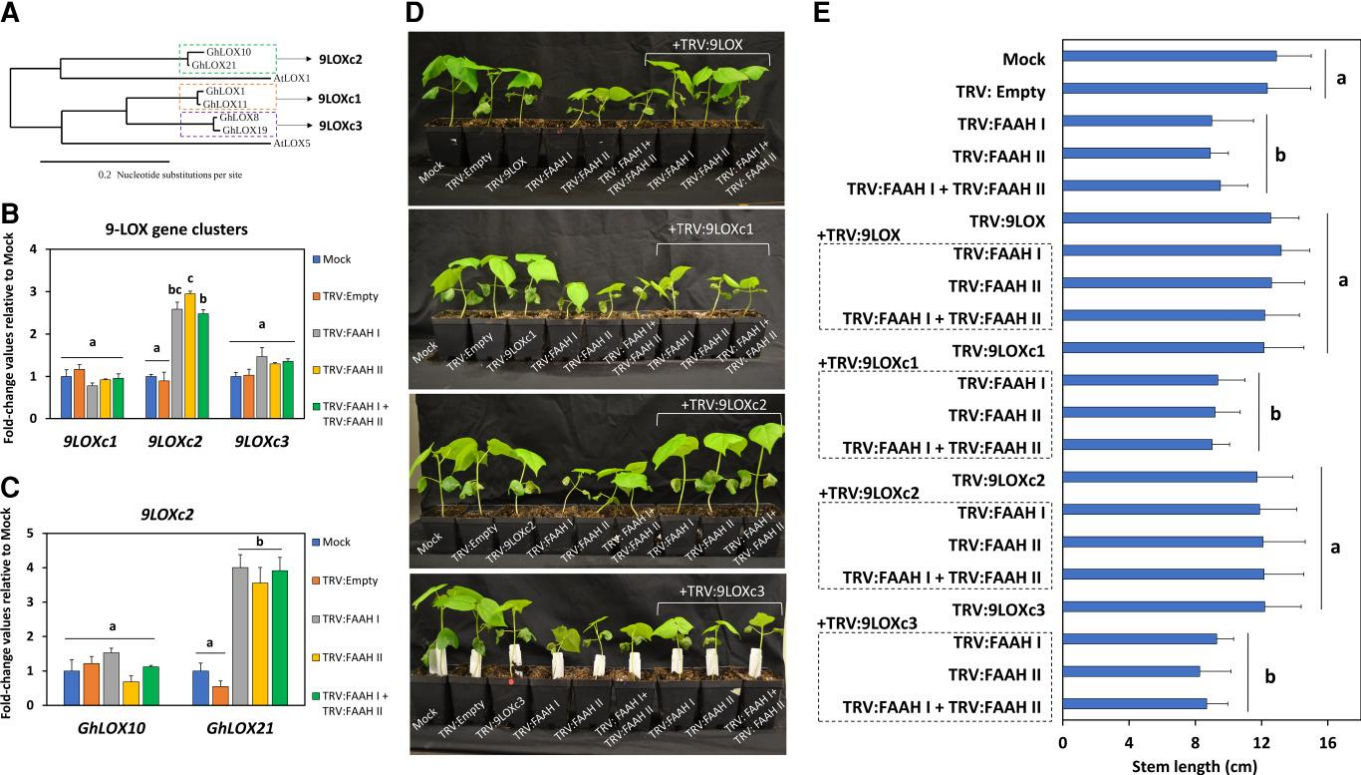
Suppression of *9-LOX*es in *FAAH* compromised cotton seedlings. A, Phylogenetic tree of *9-LOX* coding sequences. The Arabidopsis *9-LOX* genes were included as controls. Enclosed in rectangles are the three different *9-LOX* gene clusters, namely, *9LOXc1*, *9LOXc2*, and *9LOXc3*. Scale bar = 0.2 nucleotide substitutions per site. B, RT-qPCR to quantify *9LOXc1* (*GhLOX1*, *11*), 9LOXc2 (*GhLOX10*, *21*) and *9LOXc3* (*GhLOX8*, *19*) transcripts. Error bars represent the standard deviation (SD). C, RT-qPCR for *GhLOX10* and *GhLOX21*. *UBQ1* was used as the housekeeping gene of normalization. Error bars represent the SD.Calculations were made with the ddCt method. Different letters in B and C denote significant differences (*P* < 0.05, *n* = 3) by ANOVA with Tukey's post-hoc test. D, Representative images *FAAH* and/or *9-LOX* silenced seedlings at 20 days post infiltration (dpi). E, Stem length (*n* = 13) measurements were made in the VIGS-treated seedlings. Error bars represent the SD. Different letters denote significant differences (*P <* 0.05) by ANOVA with Tukey's post-hoc test.

Given the interplay of 9-LOX and FAAH modulation of NAEs in Arabidopsis (Kilaru et al., 2011; Keereetaweep et al., 2013, 2015), and the previously demonstrated FAAH- and LOX- activities in cotton microsomes (Shrestha et al., 2002), we hypothesized that silencing of *9-LOX* genes in *FAAH* silenced backgrounds could have an impact on NAE oxylipin levels and cotton seedling growth. Previously, genome-wide characterization of *LOX* genes in cotton revealed six putative *9-LOXes* (Shaban et al., 2018). In our study, they were phylogenetically grouped as follows; *9LOXc1* (*GhLOX1, GhLOX11*), *9LOXc2* (*GhLOX10*, *GhLOX21*), and *9LOXc3* (*GhLOX8*, *GhLOX19*) (Figure 2A). We evaluated the transcript abundance of the three clusters of *9-LOX* genes in seedlings inoculated exclusively with TRV: FAAH (I and/or II). Data showed that *9LOXc2* transcripts were ≈2.4- to three-fold higher in *FAAH I* and/or *II* silenced cotton seedlings (Figure 2B; *P* < 0.05, *n* = 3), whereas *9LOXc1* or *9LOXc3* gene groups were unchanged compared with the controls (Figure 2B; *P* > 0.05, *n* = 3). Also, we used gene-specific primers and RT-qPCR to specifically quantify the *9-LOXes* from *9LOXc2* that changed in *FAAH* silenced tissues, and found that *GhLOX21* but not *GhLOX10* was significantly elevated (≈four-fold) compared with mock-inoculated or empty-vector negative controls (Figure 2C; *P* < 0.05, *n* = 3). Gene-specific primers in the 5'UTR were able to distinguish between these two pairs of *LOX* transcripts (Supplemental Table 1). These results point to GhLOX21 as the potential candidate to produce 9-NAE-HODs and to cooperate specifically with FAAH-mediated pathways to modulate seedling growth.

We used VIGS to target each LOX “cluster” individually (TRV: 9LOXc1 to c3), all 9-LOXes (all three VIGS constructs delivered together = TRV: 9LOX), or co-delivered LOX suppression constructs with TRV: FAAH (I and/or II). At 20 dpi, leaf size and stem length of TRV: 9LOXc1, c2, c3, or “all 9LOX”-inoculated seedlings were unchanged compared with mock or empty-vector controls (Figure 2, D and E; *P* > 0.05, *n* = 13) (Supplemental Figure 1 and 2). Compared with controls, seedlings co-inoculated with either TRV: 9LOXc1 or TRV: 9LOXc3 along with TRV: FAAH (I and/or II) were as stunted as seedlings where either *FAAH* group were silenced on their own (Figure 2, D, and E; *P* < 0.05, *n* = 13) (Supplemental Figure 1 and 2). By contrast, the stem length and leaf size of seedlings co-silenced with TRV: 9LOXc2 or TRV: 9LOX and either TRV: FAAH (I and/or II) treatment resembled those of the mock-inoculated or empty-vector controls (Figure 2, D and E; *P* < 0.05, *n* = 13) (Supplemental Figure 1 and 2). Together, these data suggest that silencing one or both of the *9-LOXes* in the *9LOXc2* group (*GhLOX10*, *GhLOX21*) can ameliorate the reduced growth observed in *FAAH*-silenced cotton seedlings, perhaps by preventing the formation of NAE hydroxides.

Silencing of *9-LOXes* alone or in combination with co-silencing of *FAAH* genes was confirmed by RT-qPCR (Supplemental Figures 3 and 4). Primers based on conserved sequence regions were designed to detect the amounts of *9-LOX* transcripts within each *9-LOX* “cluster” (Supplemental Table 1). These RT-qPCR amplicons do not overlap with regions used for VIGS silencing, thus, avoiding off-target detection. Silencing efficiencies of *9-LOX* gene group (*9LOXc1*, *9LOXc2*, or *9LOXc3*) transcripts were between 70% and 80% across all experiments (Supplemental Figure 3; *P* < 0.05, *n* = 3), whereas suppression of *FAAH* transcripts reached 70%–90% (Supplemental Figure 4; *P* < 0.05, *n* = 3).

In comparing NAE profiles in *FAAH/LOX* silenced cotton seedlings, we found that total NAE content (unsubstituted/non-oxygenated) was somewhat elevated in tissues co-delivered with TRV: 9LOXc2 and TRV: FAAHs (Supplemental Figure 5; *P* < 0.05, *n* = 3). Data also revealed that compared with mock and empty-vector negative controls, the contents of some specific NAEs (e.g. NAE14:0 or NAE18:0) did not change in any of the *FAAH* and/or *LOX* silencing treatments. On the other hand, compared with negative controls, NAE18:2, in particular, was elevated the highest in tissues inoculated with TRV: FAAH (I and II) or TRV: 9LOXc2 and TRV: FAAH (I and II) (Supplemental Figure 6; *P* < 0.05, *n* = 3). These data suggest that silencing of *FAAH* and 9-*LOX* gene groups can lead to misregulation of certain NAE types, like NAE18:2; however, these changes did not entirely align with differences in seedling growth in co-silenced treatments, and we suspected that oxylipins derived from NAE18:2 might provide the most insights into acylethanolamide modulation of seedling growth.

**Figure 3:**
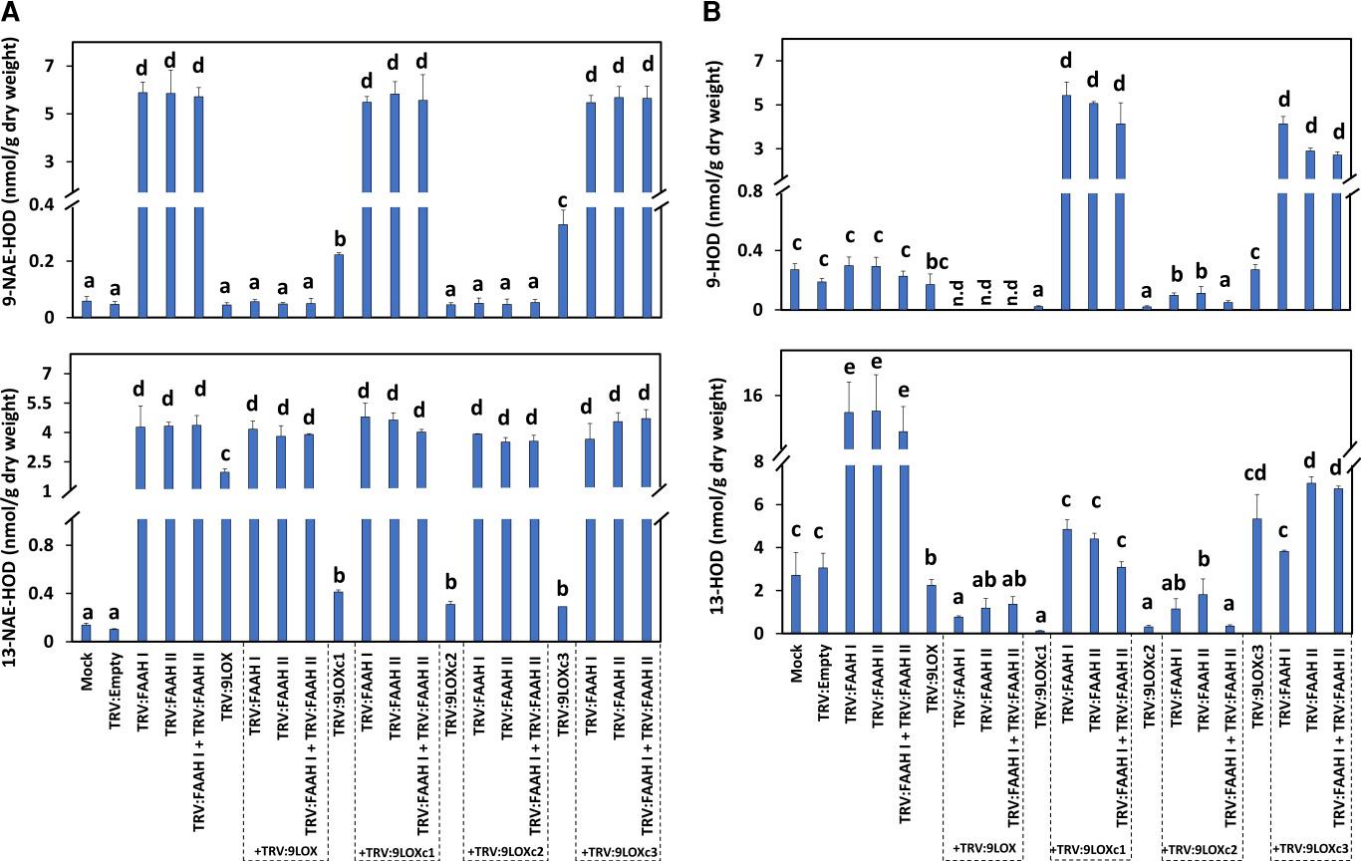
NAE- and FFA-oxylipin profiles of *FAAH* (*I* and/or *II*) and/or *9-LOX* silenced seedlings. A, 9-NAE-HOD (upper panel) and 13-NAE-HOD (lower panel) profiles. B, 9-HOD (upper panel) and 13-HOD (lower panel). Error bars in A and B represent the standard deviation (SD). Different letters in A and B denote significant differences (*P* < 0.05; *n* = 3) by ANOVA with Tukey's post-hoc test.

Quantification of NAE 18:2-derived oxylipins showed that in tissues where TRV: FAAH (I and/or II) was co-silenced with either TRV: 9LOXc2 or 9LOX, the levels of 9-NAE-HOD (Figure 3A) and seedling growth phenotypes (Figure 2D–E) were similar to those of the negative controls (Mock or TRV: Empty) (Figure 3A; upper panel*, P* < 0.05, *n* = 3). The 9-NAE-HOD content in tissues inoculated with TRV: 9LOXc2 alone (and seedling growth) was not different from mock-inoculated or empty vector controls (*P* > 0.05, *n* = 3). By stark contrast, compared with the controls, TRV: FAAH (I and/or II) co-silenced tissues with either TRV: 9LOXc1 or TRV: 9LOXc3 exhibited dramatically elevated levels of 9-NAE-HOD (≈90- to 98-fold; Figure 3A; upper panel, *P* < 0.05, *n* = 3), concomitant with reduced seedling growth (Figure 2, D, and E), similar to the TRV: FAAH-inoculated tissues alone (Figure 3A; upper panel *P* > 0.05, *n* = 3). TRV: 9LOXc1 or c3 inoculated seedlings had 9-NAE-HOD amounts that were modestly elevated (*P* < 0.05, *n* = 3) compared with negative controls but remained well below the levels found in TRV: FAAH (I and/or II) alone or their respective co-silencing with *9LOXc1* or *c3* (*P* < 0.05, *n* = 3). The amount of 13-NAE-HOD remained similarly elevated in TRV: FAAH (I and/or II) silenced or co-silenced experiments with either *9-LOX* silencing construct (Figure 3A; lower panel, *P* > 0.05, *n* = 3). In regards to FFA-oxylipin profiles, 9-HOD increased in tissues co-delivered with TRV: FAAH (I and/or II) and TRV: 9LOXc1 or c3, whereas it decreased or was undetectable in co-silencing treatments with TRV: FAAH (I and/or II) and TRV: 9LOXc2 or TRV: 9LOX (Figure 3B; upper panel*, P* < 0.05, *n* = 3). 13-HOD content only increased in TRV: FAAH (I and/or II) silenced tissues and it remained either unchanged (*P* > 0.05, *n* = 3) or reduced (*P* < 0.05, *n* = 3) across *FAAH*/*LOX* co-silencing experiments (Figure 3B; lower panel). Altogether these data support a scheme whereby FAAH (I and/or II) and the genes of a specific *9-LOX* group (*9LOXc2*) coordinately modulate cotton seedling growth via endogenous 9-NAE-HOD (but not 13-NAE-HOD) metabolite levels (Supplemental Figure 7). These data also suggest that FFA-oxylipin pools are not necessarily associated with NAE-oxylipin turnover, and are likely not associated directly with modulation of seedling growth.

### Silencing of individual *FAAH I* and *FAAH II* isoforms in cotton seedlings

**Figure 4:**
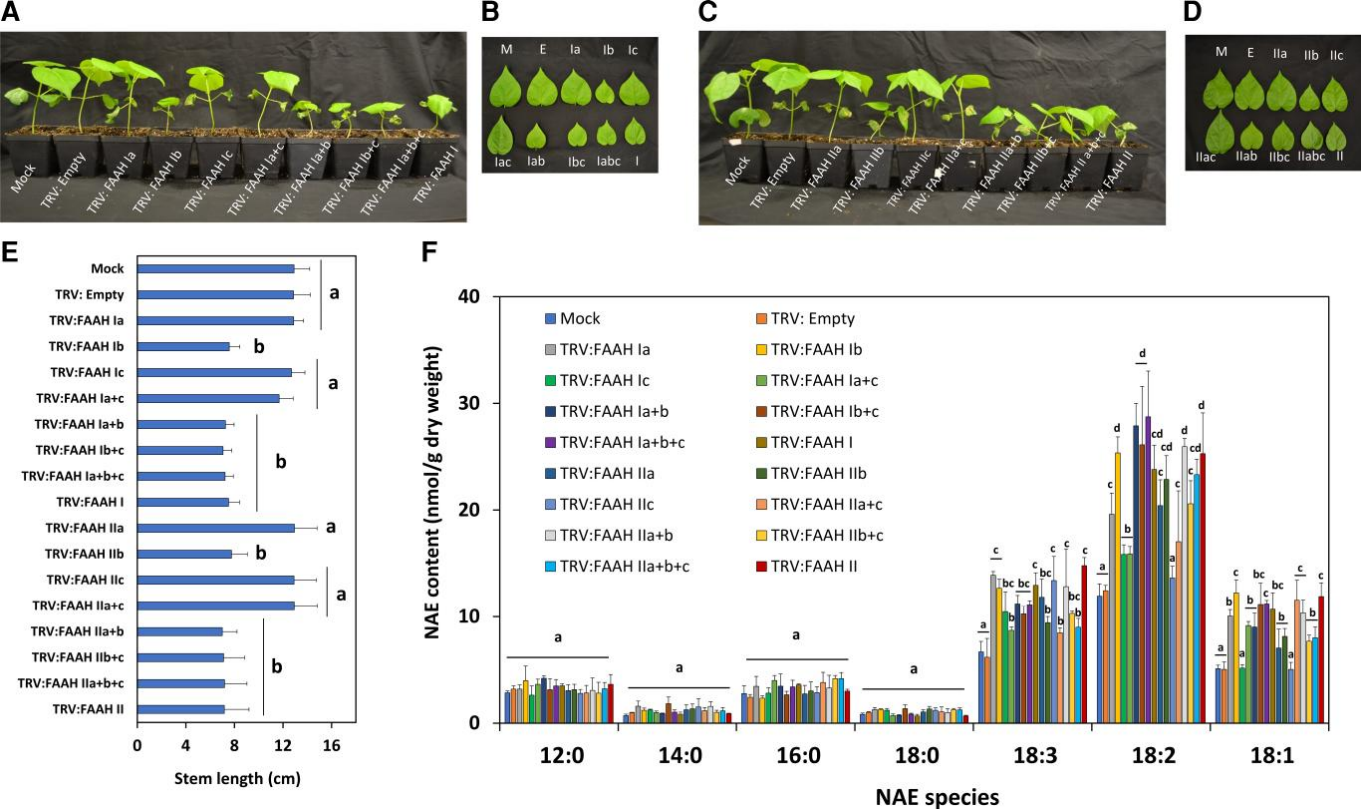
Suppression of individual cotton *FAAH I* or *II* isoforms in cotton seedlings. A, Representative images of individually silenced group I *FAAHs* (TRV: FAAH Ia, Ib, and/or Ic), and B, detached primary leaves of the same experiments. C, Representative images of individually silenced group II *FAAHs* (TRV: FAAH IIa, IIb, and/or IIc), and D, detached leaves for side-by-side comparisons. Mock (infiltration media) and TRV: Empty (vector) inoculations were used as negative controls. E, Stem length (*n* = 13) measurements made for the VIGS experiments. Error bars represent the standard deviation (SD). Different letters denote significant differences (*P* < 0.05) by ANOVA with Tukey's post-hoc test. F, Unsubstituted/non-oxygenated NAE types in *FAAH* (*Ia*, *Ib*, and/or *Ib*) or *FAAH* (*IIa*, *IIb*, and/or *IIb*) silenced tissues. Error bars represent the SD. Different letters denote significant differences (*P* < 0.05, *n* = 3) by ANOVA with Tukey's post-hoc test.

As VIGS experiments revealed growth reduction phenotypes in *FAAH I* and/or *FAAH II* silenced seedlings, this prompted us to ask whether such effect (s) could be attributed to the silencing of one or multiple cotton *FAAHs* in each group. Due to the high similarly between members of *FAAH I* or *II* genes, and to avoid off-targeting silencing, we made VIGS constructs that targeted the 5’ or 3’ UTRs of each *FAAH* (see Supplemental Table 1 for VIGS construct summaries). VIGS constructs that target individual *FAAH I* genes were named TRV: FAAH Ia, Ib, or Ic, whereas the ones targeting individual *FAAH II* were named TRV: FAAH IIa, IIb, or IIc. We delivered the constructs by themselves or in combination with other FAAH construct(s) of the same group. We included seedlings inoculated with TRV: FAAH I or TRV: FAAH II constructs in the same experiments for comparison (Figure 4), since these vectors suppressed all *FAAHs* within a given group as previously demonstrated (Figure 1). At 20 dpi, seedlings silenced with TRV: FAAH Ia and/or Ic exhibited similar seedling growth parameters (leaf size and stem length) compared with those of the mock-inoculated or empty-vector negative controls (Figure 4A, B, and E; *P* > 0.05, *n* = 13) (Supplemental Figure 8; *P* > 0.05, *n* = 13). Similar results (lack of growth effects) were observed in seedlings silenced with TRV: FAAH IIa and/or IIc (Figure 4C, D, and E; *P* > 0.05, *n* = 13) (Supplemental Figure 8; *P* > 0.05, *n* = 13). By contrast, the stem length and leaf size of seedlings inoculated with TRV: FAAH Ib by itself or in combination with any other silencing construct (TRV: FAAH Ia + Ib, TRV: FAAH Ib + Ic, TRV: FAAH Ia + Ib + Ic) led to inhibition of seedling growth (Figure 4A, B, and E; *P* < 0.05, *n* = 13). Similar results were observed in tissues inoculated with TRV: FAAH IIb by itself or in combination with any other construct (TRV: FAAH IIa + IIb, TRV: FAAH IIb + IIc, TRV: FAAH IIa + IIb + IIc) (Figure 4C, D, and E; *P* < 0.05, *n* = 13) (Supplemental Figure 8; *P* < 0.05, *n* = 13). The seedling growth inhibition outcomes observed for TRV: FAAH Ib or TRV: FAAH IIb inoculated seedlings, resembled those of the constructs which suppressed all family members of *FAAH* group I or *FAAH* group II, the TRV: FAAH I or TRV: FAAH II (*P* > 0.05). These data support the notion that either *GhFAAH Ib* or *GhFAAH IIb* are required for normal seedling growth in cotton, and that the other four *FAAH* isoforms do not participate in this specific process or stage of early seedling growth. RT-qPCR was used to assess the silencing efficiency in the experiments. *FAAH* (*Ia*, *Ib*, or *Ic*) or *FAAH* (*IIa*, *IIb*, or *IIc*) transcripts were 70%–90% lower compared with controls (Supplemental Figure 9; *P* < 0.05, *n* = 3).

Total unsubstituted/non-oxygenated NAE content was similarly elevated in tissues where either TRV: FAAH (Ia, Ib, or Ic) or TRV: FAAH (IIa, IIb, or IIc) were silenced individually or in any given combination (Supplemental Figure 10; *P* < 0.05, *n* = 3). Quantification of individual NAE types showed that the contents of NAE18:3, and NAE18:2, NAE18:1 increased in most of the *FAAH I*- or *II*-silenced tissues (Figure 4F; *P* < 0.05, *n* = 3), whereas no significant changes were observed for the saturated NAEs- NAE12:0, NAE14, NAE16:0, or NAE18:0 (Figure 4F; *P* > 0.05, *n* = 3). These data suggest that silencing of either *FAAH* (*Ia*, *Ib*, or *Ic*) or (*IIa*, *IIb*, or *IIc*) isoforms leads to elevated unsaturated 18C NAE content, specifically NAE18:2. However, these changes in unsaturated NAE profiles were modest relative to alterations in NAE18:2-derived oxylipins.

**Figure 5:**
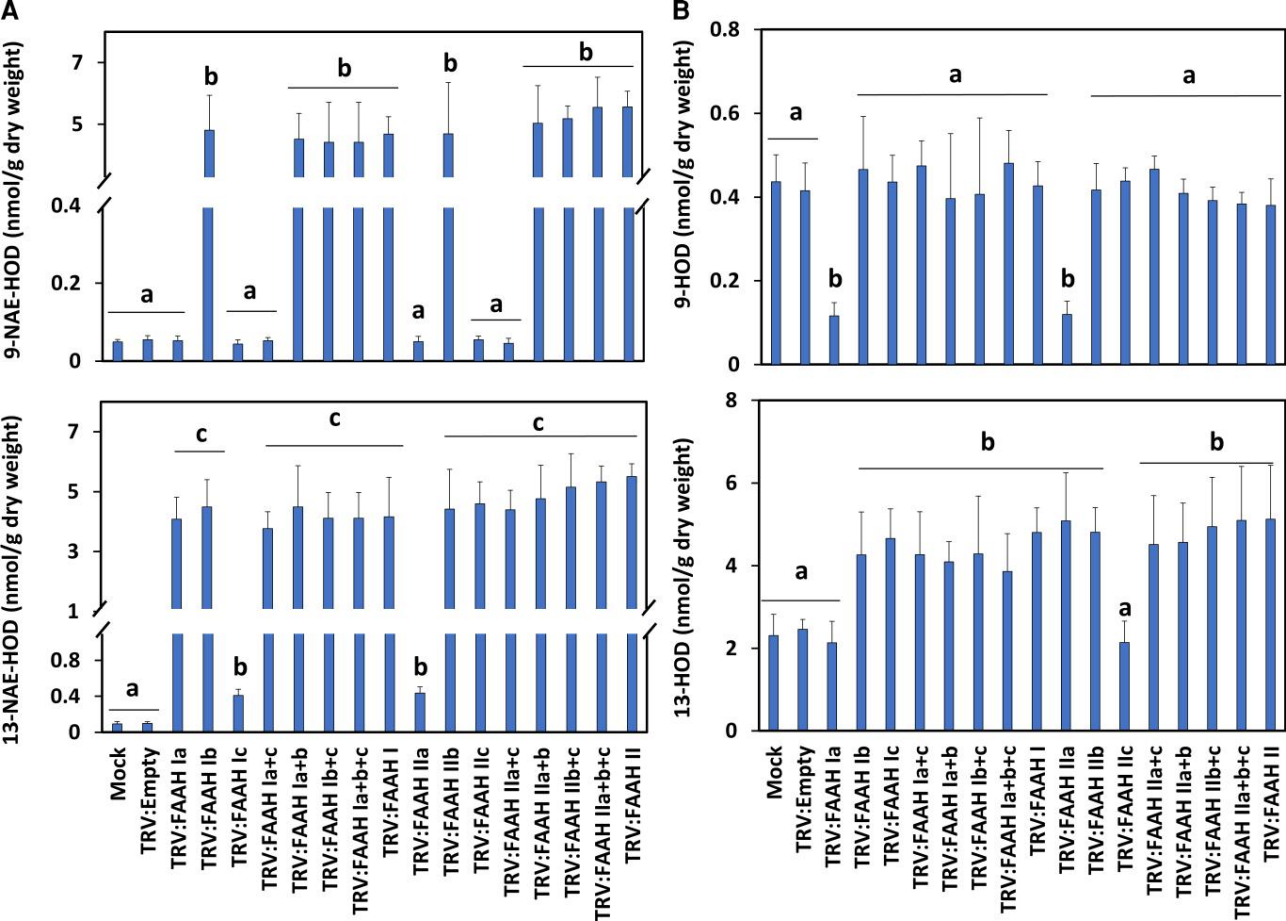
NAE- and FFA-oxylipin profiles of TRV: FAAH I (Ia, Ib, IIc) or II (IIa, IIb, IIc) inoculated seedlings. A, 9-NAE-HOD (upper panel) and 13-NAE-HOD (lower panel) profiles. B, 9-HOD (upper panel) and 13-HOD (lower panel). Error bars in A and B represent the standard deviation (SD). Different letters in A and B denote significant differences (*P* < 0.05; *n* = 3) by ANOVA with Tukey's post-hoc test.

Examination of NAE-oxylipins profiles revealed that compared with negative controls, 9-NAE-HOD increased between 96≈ to 110-fold in tissues inoculated with TRV: FAAH Ib or TRV: FAAH IIb by themselves or in combination with any other *FAAH* silencing construct of the same group (Figure 5A; upper panel, *P* < 0.05, *n* = 3). By contrast, 9-NAE-HOD remained unchanged in seedlings silenced with TRV: FAAH Ia and/or Ic, or TRV: FAAH IIa and/or IIc (Figure 5A; upper panel, *P* > 0.05, *n* = 3). These patterns of metabolite accumulation for 9-NAE-HOD exactly matched the seedling growth phenotypes (Figure 4), where seedling growth was inversely associated with 9-NAE-HOD accumulation. Growth modulation by other oxylipins was ruled out since their levels were not entirely consistent with seedling growth changes. For example, the 13-NAE-HOD content remained elevated in all *FAAH* silencing or co-silencing treatments regardless of seeding growth inhibition, with somewhat lower levels in TRV: FAAH Ic- or TRV: FAAH IIa-treated seedlings (Figure 5A; lower panel*, P* < 0.05, *n* = 3). FFA-oxylipin profiles showed that 9-HOD content did not change in most of the treatments tested (Figure 5B; upper panel, *P* > 0.05, *n* = 3) with the exception of TRV: FAAH Ia or IIc (*P* < 0.05, *n* = 3). And the 13-HOD levels were significantly elevated in most treatments (Figure 5B; lower panel, *P* < 0.05, *n* = 3) with the exception of TRV: FAAH Ia or IIc (*P* > 0.05, *n* = 3). Altogether, these data suggest that the metabolism of endogenous 9-NAE-HOD (but not 13-NAE-HOD) by GhFAAH Ib or GhFAAH IIb is strongly linked with growth modulation of cotton seedlings, and also points to participation of other, additional pathways that can influence 13-HOD or 9-HOD pools.

### Silencing of *GhLOX10* or *GhLOX21* in FAAH-compromised cotton seedlings

**Figure 6:**
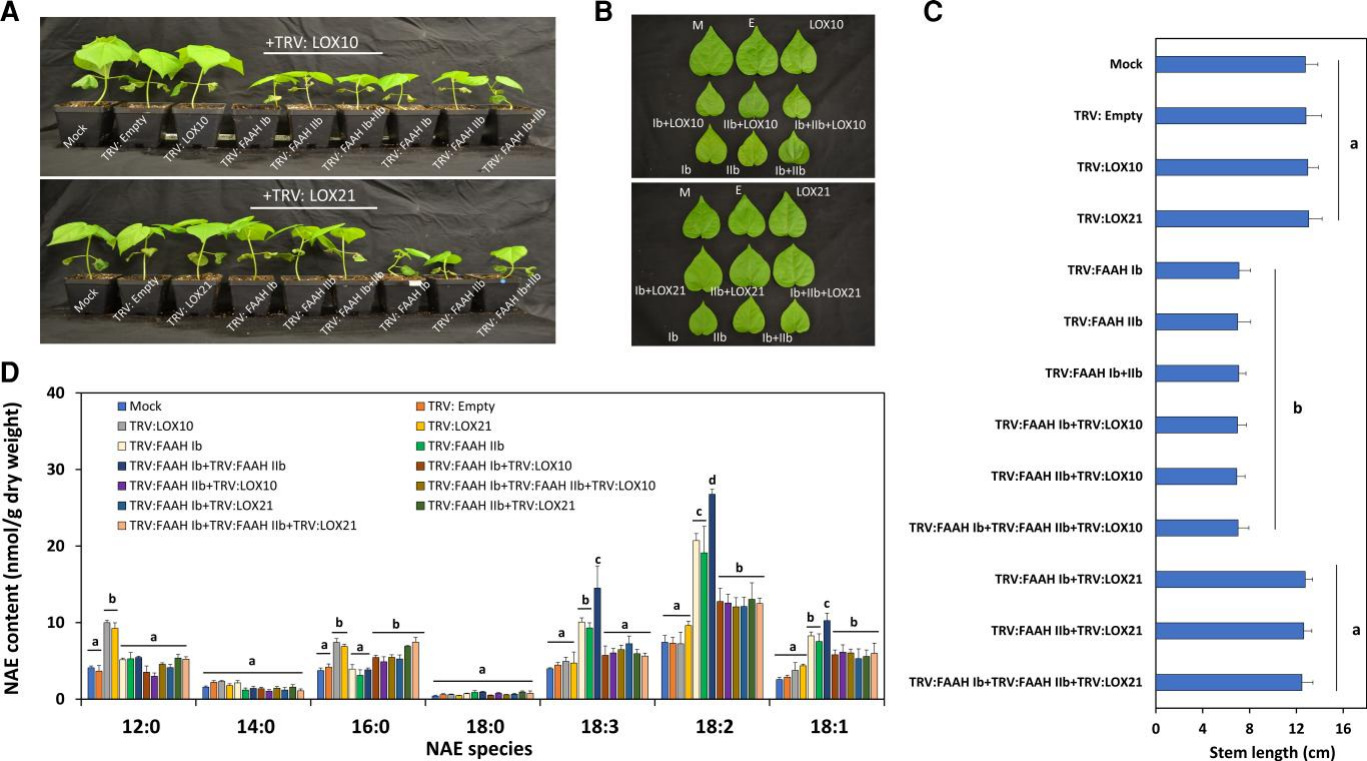
Suppression of *GhLOX* (*10* or *21*) and/or *GhFAAH* (*Ib* and/or *IIb*) in cotton seedlings. A, Representative images of whole seedlings at 20 dpi. B, Detached leaves from VIGS experiments. Abbreviations: M, Mock; E,TRV: Empty; LOX10= TRV: LOX10; Ib + LOX10= TRV: FAAH Ib + TRV: LOX10; IIb + LOX10= TRV: FAAH IIb + TRV: LOX10; Ib + IIb + LOX10= TRV: FAAH Ib + TRV: FAAH IIb + TRV: LOX10; LOX21= TRV: LOX21; Ib + LOX21= TRV: FAAH Ib + TRV: LOX21; IIb + LOX21= TRV: FAAH IIb + TRV: LOX21; Ib + IIb + LOX21= TRV: FAAH Ib + TRV: FAAH IIb + TRV: LOX21. C, Stem length (*n* = 13) measurements for VIGS experiments. Error bars represent the standard deviation (SD). Different letters denote significant differences (*P* < 0.05) by ANOVA with Tukey's post-hoc test. D, Unsubstituted/non-oxygenated NAE types in *FAAH* (*Ib*, and/or *IIb*) and/or *9LOXc2* (*GhLOX10*, or *GhLOX21*) silenced tissues. Error bars represent the SD. Different letters in C and D denote significant differences (*P* < 0.05, *n* = 3) by ANOVA with Tukey's post-hoc test.

The growth restoration observed in seedlings inoculated with TRV: 9LOXc2 and TRV: FAAH (I and/or II) gene groups prompted us to investigate whether such effect can be attributed to one or two of the genes that composed the *9LOXc2* “cluster” (*GhFAAH10*, *GhLOX21*). As the previously described experiments were able to narrow down the number of FAAH isoforms associated with growth from six to two, namely, *GhFAAH Ib* and *GhFAAH IIb*, we decided to carry out an experiment in which TRV: FAAH (Ib and/or IIb) were co-inoculated with silencing constructs that can separately target *GhLOX10* (TRV: LOX10) or *GhLOX21* (TRV: LOX21) (Figure 6). At 20 dpi, compared with mock or empty-vector controls, seedlings co-silenced with TRV: LOX10 and TRV: FAAH (Ib and/or IIb) were as stunted (shorter stem length and the leaf size) as the ones treated with TRV: FAAH Ib and/or FAAH IIb alone (Figure 6, A–C; *P* < 0.05, *n* = 13) (Supplemental Figure 11; *P* < 0.05, *n* = 13). By contrast, seedlings co-silenced with TRV: LOX21 and TRV: FAAH (Ib and/or IIb) had no evident changes in their growth phenotypes (Figure 6, A–C; *P* > 0.05, *n* = 13) (Supplemental Figure 11; *P* > 0.05, *n* = 13). The growth of seedlings inoculated with TRV: GhLOX10 or TRV: GhLOX21 by themselves did not exhibit changes in growth when compared with controls (Figure 6, A–C; *P* > 0.05, *n* = 13) (Supplemental Figure 11; *P* > 0.05, *n* = 13). Altogether, these data suggest that both GhFAAH Ib and IIb are key factors in seedling growth, and that silencing of *GhLOX21* was sufficient to restore FAAH-mediated seedling growth inhibition.

RT-qPCR was performed to assess silencing efficiency, data shown that 80%–90% of *GhFAAH Ib* or *GhFAAH IIb* transcripts were reduced in either one of the silencing or co-silencing experiments (Supplemental Figure 12A; *P* < 0.05, *n* = 3). Similarly, 70%–90% of *GhLOX10* or *GhLOX21* were reduced in silencing or co-silencing experiments (Supplemental Figure 12B; *P* < 0.05, *n* = 3). In addition, we evaluated the amount of *GhLOX10* or *GhLOX21* in *GhFAAH Ib* and/or *GhFAAH IIb* silenced cotton seedlings. Data showed that TRV: FAAH (I and/or IIb) treated plants had ≈three- to four-fold higher levels of *GhLOX21* than in controls (Supplemental Figure 12B; *P* < 0.05, *n* = 3). Conversely, in the same tissues, no significant changes were detected for *GhLOX10* (Supplemental Figure 12B; *P* > 0.05, *n* = 3). These data suggest that *GhLOX21* rather than *GhLOX10* is upregulated in *FAAH Ib* and/or *IIb* suppressed cotton seedlings.

NAE profiles showed elevated total unsubstituted/non-oxygenated NAE contents in all silencing experiments with TRV: FAAH (Ib and/or IIb) and/or TRV: LOX (10 or 21) compared with controls (Supplemental Figure 13; *P* < 0.05, *n* = 3). Close inspection of the NAE profiles showed that the contents of NAE18:2, and NAE18:1 were significantly elevated in the same treatments compared with controls. Also, delivery of TRV: LOX10 or TRV: LOX21 by themselves resulted in elevated amounts of some saturated NAEs (e.g. NAE12:0) when compared with controls (Figure 6D; *P* < 0.05, *n* = 3). On the other hand, NAEs like NAE14:0 or NAE18:0 were unchanged in either one of the treatments (Figure 6D; *P* > 0.05, *n* = 3). These data suggest that silencing of either *FAAH* and/or *9LOXc2* isoforms can lead to misregulation of certain NAEs, and, especially when *FAAH* is silenced, unsaturated 18C NAEs were the principal NAEs that were elevated. However, these changes in levels of unsubstituted NAEs were of considerably less magnitude than the changes in NAE hydroxides.

**Figure 7:**
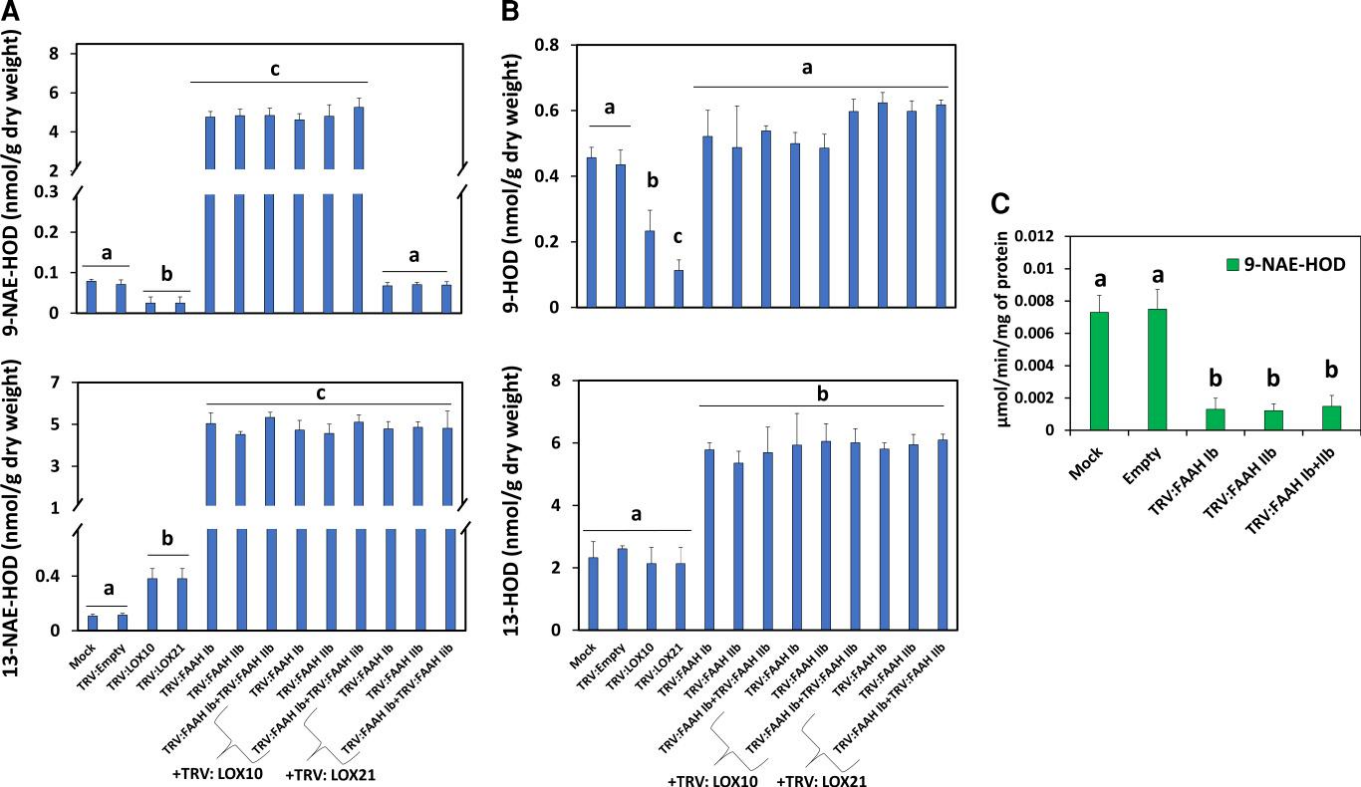
NAE- and FFA-oxylipin profiles of *GhLOX* (*10* or *21*) and/or *GhFAAH* (*I* and/or *II*) silenced seedlings. A, 9-NAE-HOD (upper panel) and 13-NAE-HOD (lower panel) profiles. B, 9-HOD (upper panel) and 13-HOD (lower panel) profiles. Error bars in A and B represent the standard deviation (SD). Different letters in A and B denote significant differences (*P* < 0.05; *n* = 3) by ANOVA with Tukey's post-hoc test. C, FAAH activity assay with plant homogenates (100 µg of protein) from *FAAH* (*I* and/or *II*) silenced plants. Error bars represent the SD. Different letters denote significant differences (*P* < 0.05; *n* = 4) by ANOVA with Tukey's post-hoc test.

As in previous treatments, it was the changes in NAE18:2-derived oxylipins that changed most dramatically in *FAAH*-silenced plants, whilst the co-silencing of *GhLOX21*, but not *GhLOX10*, nearly eliminated the accumulation of 9-NAE-HOD (Figure 7). Indeed, quantification of NAE-oxylipins revealed that 9-NAE-HOD levels in seedlings inoculated with TRV: LOX10 and TRV: FAAH (Ib and/or IIb) were ≈64 to 75-fold higher than controls (Figure 7A; upper panel, *P* < 0.05, *n* = 3), and this increase in 9-NAE-HOD content was comparable to that of TRV: FAAH (Ib and/or IIb)-only inoculated seedlings. Conversely, 9-NAE-HOD levels were much lower in seedlings co-inoculated with TRV: LOX21 and TRV: FAAH (Ib and/or IIb), whereas 13-NAE-HOD remained elevated in all silencing experimental treatments when compared with unsilenced controls (Figure 7A; lower panel, *P* < 0.05, *n* = 3). Notably, the contents of 9-NAE-HOD or 13-NAE-HOD in tissues where TRV: LOX10 or TRV: LOX21 were delivered by themselves, were respectively lower or greater than controls (Figure 7A; upper and lower panels, *P* < 0.05, *n* = 3). Quantification of FFA-oxylipins showed that compared with non-silenced controls, 9-HOD was lower in TRV: LOX10 or TRV: LOX21 treated seedlings (Figure 7B; upper panel, *P* < 0.05, *n* = 3), whereas the same lipid remained unchanged for any other silencing treatments (*P* > 0.05). 13-HOD, compared with controls, remained unchanged in TRV: LOX10 or TRV: LOX21 samples (Figure 7B; lower panel, *P* < 0.05, *n* = 3), whereas it was significantly elevated in other treatments (*P* < 0.05). Altogether, these data helped to define a complex scheme in which GhFAAH Ib, GhFAAH IIb, and GhLOX21 cooperate to support a balance of 9-NAE-HOD which likely is tuned to support the rate of seedling growth (Supplemental Figure 14).

### FAAH activity assays in *FAAH Ib* and *FAAH IIb* silenced seedlings

To confirm that FAAH activity in cotton was lower in *GhFAAH Ib* and/or *IIb* silenced seedlings, and that this was concomitant with both cotton growth reduction and a decline in the content of endogenous 9-NAE-HOD, fluorescent-based assays were used to quantify the amount of ethanolamine produced from exogenously added 9-NAE-HOD into cell-free homogenate fractions (protein source) derived from TRV: FAAH (Ib and/or IIb) inoculated seedlings. The rate of amidohydrolase activity in either one of the *FAAH* silencing treatments declined ≈85% when compared with homogenates of either mock or TRV: Empty controls (Figure 7C; *P* < 0.05, *n* = 4). These data suggest that GhFAAH Ib and IIb participate in the hydrolysis of GhLOX21-generated 9-NAE-HOD. Further, these data also support our findings that these two *FAAH* genes modulate endogenous 9-NAE-HOD accumulation *in planta*, and their ultimate association with seedling growth.

### Exogenous applications of NAE-oxylipins and FFA-oxylipins in cotton seedlings

**Figure 8:**
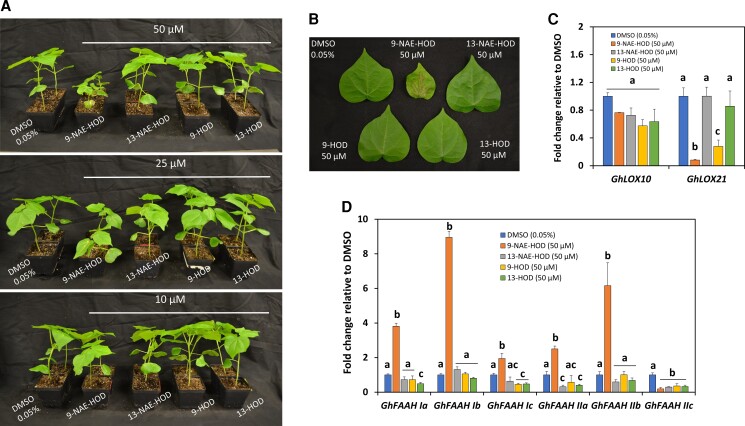
Exogenous applications of NAE- and FFA-oxylipins. A, Representative images of 9-NAE-HOD-, 13-NAE-HOD-, 9-HOD-, or 13-HOD- irrigated cotton seedlings at 10, 25, and 50 µM. B, Detached primary leaves from 50 µM treatments. C, RT-qPCR to measure *GhLOX10*, *GhLOX21*, (D) *FAAH I* and *FAAH II* transcripts in NAE- or FFA-oxylipin treated seedlings. *UBQ1* was used as the housekeeping gene of normalization. Error bars in C and D represent the standard deviation (SD). Calculations were made with the ddCt method. Different letters in C and D denote significant differences (*P* < 0.05, *n* = 3) by ANOVA with Tukey's post-hoc test.

To corroborate genetic and biochemical experiments, we assessed the direct impact of NAE- or FFA- oxylipins in cotton seedling growth by irrigating 10-day-old cotton (Coker 312) seedlings with 9-NAE-HOD, 13-NAE-HOD, 9-HOD, or 13-HOD. Seedling growth phenotypes were assessed after 18 days of initiating these pharmacological treatments (Figure 8). Compared with solvent-only control 0.05% DMSO (v/v in ultrapure water), no obvious growth effects were observed in seedlings treated with 50 µM of 13-NAE-HOD, 9-HOD, or 13-HOD, whereas seedlings irrigated with 50 µM of 9-NAE-HOD and their associated leaves were severely stunted (Figure 8A–B). At 25 µM, 9-NAE-HOD-induced inhibition was less noticeable, and at 10 µM minimal changes in seedling growth were observed (Figure 8A). Thus, these data suggest that 9-NAE-HOD, and not 13-NAE-HOD or their FFA-oxylipins, confers a dose-dependent inhibition in cotton seedling growth, reinforcing conclusions derived from VIGS experiments.

RT-qPCR was used to assess *GhLOX10*, *GhLOX21*, *GhFAAH I*, or *GhFAAH II* transcript changes in tissues fed with 50 µM of NAE- or FFA-oxylipins. Compared with controls, *GhLOX10* remained unchanged (*P* > 0.05, *n* = 3) in all treatments whilst *GhLOX21* was downregulated in 9-NAE-HOD- or 9-HOD-irrigated seedlings (Figure 8C, *P* < 0.05, *n* = 3). *GhFAAH IIb* and *GhFAAH Ib* transcripts in 9-NAE-HOD-treated seedlings were between ≈six- and nine-fold higher than controls (Figure 8D, *P* < 0.05, *n* = 3). In the same tissues, *GhFAAH Ia*, *Ic*, or *IIa* were between ≈two- and three-fold greater than controls (Figure 8D, *P* < 0.05, *n* = 3). *GhFAAH IIc* remained unchanged in 9-NAE-HOD-treated seedlings (*P* > 0.05, *n* = 3). The transcripts of certain *FAAH I* or *FAAH II* genes declined following irrigation with 13-NAE-HOD, 9-HOD, or 13-HOD (Figure 8D, *P* < 0.05, *n* = 3). These data revealed that applications of 9-NAE-HOD in cotton seedlings resulted in upregulation of most endogenous cotton FAAHs, especially *GhFAAH Ib* and *GhFAAH IIb*. Also, downregulation of *GhLOX21* may indicate a negative feedback mechanism triggered by elevated levels of 9-NAE-HOD and/or its corresponding FFA-HOD (9-HOD).

### Silencing of *GhLOX21* in cotton seedlings overexpressing Arabidopsis *FAAH*

**Figure 9:**
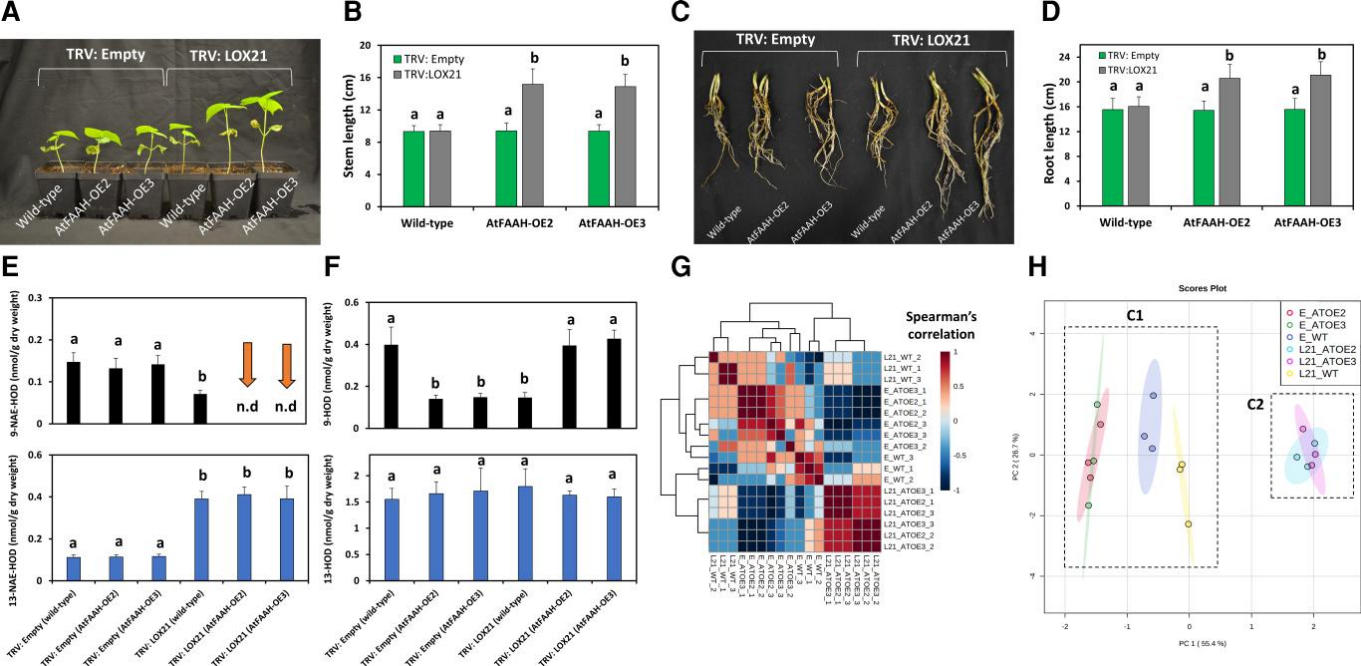
Suppression of *GhLOX21* in non-transgenic (wild-type) and *AtFAAH*-overexpressing transgenic cotton seedlings. A, Representative images of *GhLOX21* silenced wild-type or AtFAAH-OE whole seedlings. B, Stem length measurements (*n* = 8). C, Detached roots. D, Root length measurements (*n* = 8). E, NAE- and F, FFA-oxylipin profiles. Error bars in B, D, E, and F represent the standard deviation (SD). Different letters in B, D, E, and F denote significant differences (*P* < 0.05; *n* = 3) by ANOVA with Tukey's post-hoc test. G, Spearman's correlation ranking and (H, principal component analysis (PCA) with oxylipin data sets. Abbreviations: E_WT_(1–3) = wild-type infiltrated with TRV: Empty (replicates 1–3); E_ATOE2_(1–3) = AtFAAH-overexpressing line 2 infiltrated with TRV: Empty (replicates 1–3); E_ATOE3_(1–3) = AtFAAH-overexpressing line 3 infiltrated with TRV: Empty (replicates 1–3); L21_WT_(1–3) = wild-type infiltrated with TRV: LOX21 (replicates 1–3); L21_ATOE2_(1–3) = AtFAAH-overexpressing line 2 infiltrated with TRV: LOX21 (replicates 1–3); L21_ATOE3_(1–3) = AtFAAH-overexpressing line 3 infiltrated with TRV: LOX21 (replicates 1–3).

We hypothesized that silencing of *GhLOX21* in cotton lines overexpressing the transgene *AtFAAH* (AtFAAH-OE) may substantially reduce or eliminate accumulation of endogenous 9-NAE-HOD, and that this, in turn, could lead to enhanced seedling growth. We used VIGS to silence *GhLOX21* in two previously characterized cotton transgenic lines, namely AtFAAH-OE2 and AtFAAH-OE3 (Arias-Gaguancela et al., 2022), and utilized non-transgenic (Coker 312- “wild-type”) cotton seedlings as controls. Data revealed that the stems and roots of TRV: LOX21 (AtFAAH-OE lines) were longer compared with either TRV: LOX21 (wild-type) or TRV: Empty (wild-type or AtFAAH-OE lines) seedlings (Figure 9A–D, *P* < 0.05, *n* = 8). These data suggest that silencing of *GhLOX21* in cotton seedlings with ectopic expression of *AtFAAH* results in enhanced seedling growth beyond that observed with endogenous levels of *FAAH* expression.

Oxylipin quantification in the same tissues revealed that compared with TRV: Empty (wild-type or AtFAAH-OE lines), endogenous 9-NAE-HOD was undetectable in TRV: LOX21 (AtFAAH-OE2 or OE3) lines (Figure 9E; upper panel *P* < 0.05, *n* = 3). On the other hand, compared with TRV: Empty (wild-type or AtFAAH-OE lines), endogenous 13-NAE-HOD was ≈four-fold higher in TRV: LOX21 (wild-type or AtFAAH-OE lines) (Figure 9E; lower panel *P* < 0.05, *n* = 3). The FFA-oxylipin, 9-HOD decreased in TRV: Empty (AtFAAH-OE lines) or TRV: LOX21 (wild-type) when compared with any other treatment (Figure 9F; upper panel *P* < 0.05, *n* = 3). No changes in 13-HOD were detected in any of the silencing treatments made in either wild-type or AtFAAH-OE lines (Figure 9F; lower panel *P* > 0.05, *n* = 3). Spearman's rank correlation was conducted with NAE- and FFA-oxylipin data sets (Figure 9G). Data support a positive strong correlation between TRV: LOX21 (AtFAAH-OE lines) samples, and a negative correlation with respect to TRV: LOX21 (wild-type) or TRV: Empty (wild-type or AtFAAH-OE lines) oxylipin data sets (Figure 9G). Also, principal component analysis (PCA) with the same data revealed two major clusters or groups (C1 and C2). C1 comprised by TRV: LOX21 (wild-type) or TRV: Empty (wild-type or AtFAAH-OE lines) was separated from C2, which is composed by TRV: LOX21 in both AtFAAH-OE lines (Figure 9H). Altogether, these analyses support the notion that silencing of *GhLOX21* in cotton seedlings overexpressing *AtFAAH* leads to distinctive, predictable changes in NAE oxylipin content and seedling growth. Further, these results suggest that targeting a reduction in NAE-9-HOD levels, either through FAAH or LOX isoforms could improve rates of seedling growth and development in this crop species.

## Discussion

NAEs have been shown to be metabolized by competing FAAH and LOX pathways (Shrestha et al., 2002; Kilaru et al., 2011; Keereetaweep et al., 2013, 2015). NAE turnover is considered to participate in multiple physiological processes in the model plant Arabidopsis (Wang et al., 2006b; Teaster et al., 2007, 2012; Cotter et al., 2011). However, it was recently revealed through structural and phylogenetic comparisons that additional FAAH isoforms exist in angiosperms, and *FAAHs* are expanded into two distinct groups in genomes outside of Arabidopsis and Brassicaceae (Aziz & Chapman, 2020). In cotton, the FAAH gene family is represented by three isoforms in each group for a total of six *FAAH* genes (Arias-Gaguancela et al., 2022). Compared with the single *FAAH* gene in Arabidopsis, the situation in cotton is much more complex with additional *FAAH* genes that potentially could represent additional physiological relevance.

**Figure 10:**
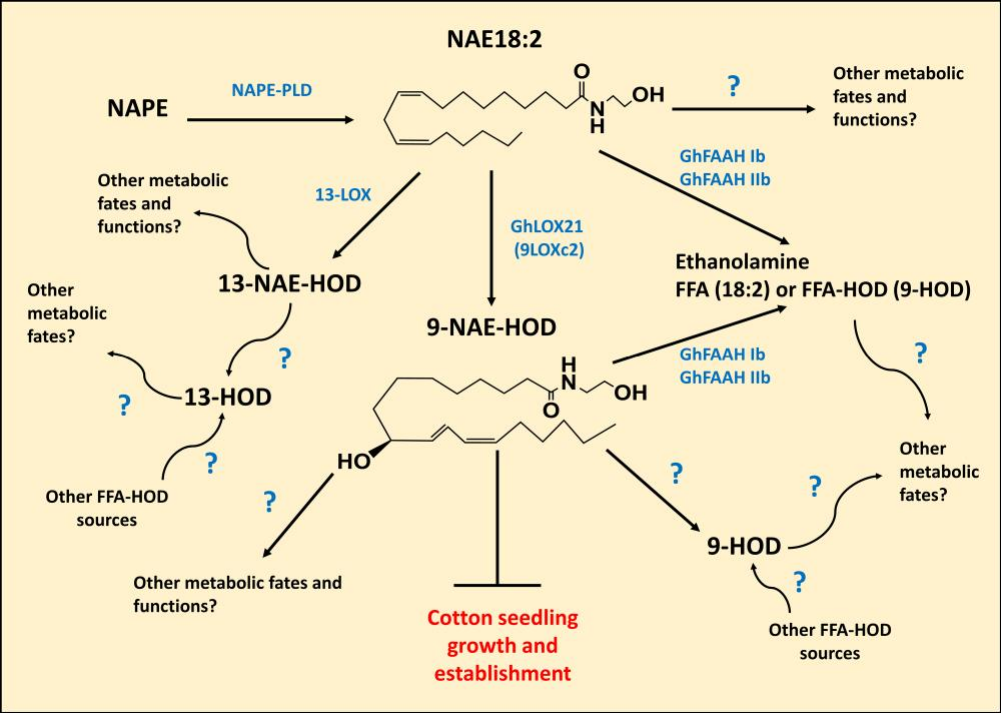
Simplified model showing the enzyme and metabolites that could participate in 9-NAE-HOD metabolism. NAPE converts NAPE into NAE18:2. Later, this NAE can be subjected to hydrolysis by FAAH (GhFAAH Ib/IIb) into ethanolamine and free fatty acids, oxidation by 9-LOX (GhLOX21) or 13-LOX to yield 9-NAE-HOD or 13-NAE-HOD, or be processed by other yet to be determined mechanisms. 13-NAE-HOD downstream processing into 13-HOD could be performed by other undetermined pathways, whereas 9-NAE-HOD could be processed into 9-HOD by either FAAH Ib and/or IIb, or other means. Data presented in this study support the notion that 9-NAE-HOD negatively affects cotton seedling development. Question marks represent unknown metabolic steps, arrows indicate the direction of the reaction, and blunt end lines represent inhibition or blockage. Abbreviations: NAPE, *N*-acylphosphatidylethanolamine; NAPE-PLD, *N*-acylphosphatidylethanolamine - phospholipase D; NAE18:2, (9*Z*,12*Z*)-*N*-(2-hydroxyethyl)octadeca-9,12-dienamide; 9-NAE-HOD, (9*S*,12*Z*,10*E*)-9-hydroxy-10,12-octadecadienoylethanolamide; 13-NAE-HOD, (13*S*,9*Z*,11*E*)-13-hydroxy-9,11-octadecadienoylethanolamide; 9-HOD, (9*S*,10*E*,12*Z*)-9-Hydroxyoctadeca-10,12-dienoic acid; 13-HOD, (9*Z*,11*E*,13*S*)-13-Hydroxyoctadeca-9,11-dienoic acid; FFA, free fatty acid; FFA-HOD, free fatty acid hydroxide; 13-LOX, 13-lipoxygenase; GhLOX21, *Gossypium hirsutum* lipoxygenase 21; 9LOXc2, 9-lipoxygenase cluster 2; GhFAAH Ib, *Gossypium hirsutum* fatty acid amide hydrolase Ib; GhFAAH IIb, *Gossypium hirsutum* fatty acid amide hydrolase IIb.

FAAH and its cooperation with LOX pathways have not been extensively studied in plants other than Arabidopsis, and here we provide evidence that two out of the six cotton FAAHs (GhFAAH Ib and GhFAAH IIb) and one of six 9-LOXes (GhLOX21) modulate the homeostasis of the endogenous acylethanolamide oxylipin, 9-NAE-HOD, during seedling development (Figure 10). In our experiments, an increase in endogenous 9-NAE-HOD in *FAAH* (*Ib* and/or *IIb*) silenced seedlings coincided with both a reduction in cotton seedling growth and a reduction of amidohydrolase activity. Pharmacological assays corroborated the genetic experiments, and showed that 9-NAE-HOD selectively inhibited growth when applied exogenously. We also showed that silencing of *GhLOX21* in cotton lines that were overexpressing the transgene *AtFAAH* resulted in both an increase in seedling growth and depletion of detectable internal 9-NAE-HOD content. The selective and potent activity of 9-LOX-derived 9-NAE-HOD is reminiscent of its participation in secondary dormancy in Arabidopsis where 9-NAE-HOD accumulated and interacted with ABA signaling to arrest seedling development (Keereetaweep et al., 2015). In Arabidopsis, the action of NAE oxylipins appeared to be restricted to a very early window of seedling development, and these lipids were inactive in true leaves of older seedlings (Keereetaweep et al., 2013, 2015). So, although not exactly overlapping in physiological context, it seems that seedling growth in cotton also is modulated through an interplay acylethanolamide oxidation and hydrolysis, a process which has been conserved despite elaboration of both *FAAH* and *LOX* gene families. Four other *FAAH* genes were not involved in seedling growth regulation, suggesting that there may be additional functions for this *FAAH* gene family, and this remains to be explored further. Indeed, there was a report that NAE signaling was involved in *Verticillium dahliae* pathogenesis in cotton plants and that this invoked expression of several cotton *FAAH* genes (Zhang et al., 2019). The association between seedling physiology and NAE metabolism via specific FAAHs and/or 9-LOXes could provide novel tools for future agricultural applications. Given that elevated levels of 9-NAE-HOD led to reduced seedling growth, we hypothesized that a reduction of 9-NAE-HOD would lead to enhanced seedling growth. The silencing of *GhLOX21* in plants led to a reduction of 9-NAE-HOD levels, but this was not accompanied by significant increases in seedling growth compared with non-silenced plants (Figures 6 and 7). To drive 9-NAE-HOD levels lower, we tested the silencing of *GhLOX21* under conditions of increased FAAH capacity, in two cotton lines that were overexpressing the transgene *AtFAAH*. As might be anticipated, this led to a further reduction in 9-NAE-HOD levels (below detection in seedling tissues) and a marked overall increase in seedling growth compared with non-silenced controls. Hence an interplay between hydrolysis and the formation of 9-NAE-HOD can be dramatically shifted, and this can lead to predictable differences in seedling growth. Raising the capacity of FAAH activity has been shown to enhance plant growth elsewhere in Arabidopsis (Wang et al., 2006a) and more recently in cotton seedlings (Arias-Gaguancela et al., 2022). Previously, cotton seedlings overexpressing *AtFAAH* that were fed with a chemical enhancer of FAAH activity, 3-*n*-pentadecylphenol-ethanolamide, had reduced levels of multiple NAEs, and especially no detectable 9-NAE-HOD. These “hyper-activated” *FAAH* OE cotton seedlings exhibited accelerated growth (Arias-Gaguancela et al., 2022), similar to the *GhLOX21*-silenced *AtFAAH*-overexpressing plants here (Figure 9), most likely due to a depleted 9-NAE-HOD by FAAH. The stepwise silencing of *FAAHs* in cotton, first in overall groups I and II, and ultimately with individual FAAH-specific VIGS constructs, led to the conclusion that both *GhFAAH Ib* and *GhFAAH IIb* were required for normal seedling growth. Silencing both genes together did not generate an additive effect, as might be anticipated if these isoforms acted independently. Consequently, it may be that these gene products act together in a dependent manner, so that without either component, growth is reduced. Indeed, silencing either *FAAH* isoform resulted in similar levels of 9-NAE-HOD compared with silencing of both isoforms together. For Arabidopsis, FAAH was structurally determined, by X-ray crystallography, to be organized as a homodimer (Aziz et al., 2019; Aziz & Chapman, 2020). In Arabidopsis, there is only one *FAAH* gene (defined as group I). There is no structural information at this time for group II FAAHs, and perhaps in plants with multiple FAAH isoforms, these FAAH proteins might organize as heterodimers (or larger hetero-oligomers) and both subunits are required for a fully active FAAH complex. Such a scenario remains to be investigated, but it would be consistent with the growth phenotypes, endogenous 9-NAE-HOD levels and with residual enzyme activities in *GhFAAH Ib*- and *GhFAAH IIb*- silenced plants.

Although the pattern of endogenous accumulation of 9-NAE-HOD was the only oxylipin metabolite that invariably was inversely associated with growth inhibition, we wanted to compare the direct effects of other potential NAE 18:2-derived acylethanolamide- oxylipins (9-NAE-HOD or 13-NAE-HOD) or FFA- oxylipins (9-HOD or 13-HOD). Similar to results in Arabidopsis (Keereetaweep et al., 2015), only the applied 9-NAE-HOD had an effect on growth, while all of the others were inactive in this seedling growth stage (Figure 8). In the cotton genome, there are 21 putative lipoxygenase genes distributed as follows; *9-LOX* (six genes), *13-LOX* type I (four genes), and *13-LOX* type II (11 genes) (Shaban et al., 2018). We focused our attention on the predicted *9-LOX* genes, and used the VIGS approach in a stepwise manner to identify the *9-LOX* gene that was responsible for both 9-NAE-HOD accumulation and the associated inhibition of seedling growth (Figures 2 and 3 and 6 and 7). Certainly, the many other LOX genes are likely to play an array of physiological roles, but it appeared that only *GhLOX21* works in cooperation with *GhFAAH* isoforms to modulate ethanolamide oxylipin levels and seedling growth. Although only speculation at this point, there may be additional isoform interactions between the other *GhFAAH* and *GhLOX* genes to influence the metabolism of additional acylethanolamide oxylipins (like 13-LOX-derived metabolites) in other conditions or stages of cotton plant development.

The NAE products of 13-LOX (13-NAE-HOD or 13-HOD) had no evident effect in growth when applied exogenously. However, their markedly elevated contents in *FAAH* and/or *9-LOX* silenced plants may have some unnoticed downstream impacts. It may be possible that NAE products of 13-LOXes could be associated with various stress signaling responses that might not have been evident in our studies of seedling growth. For example, in upland cotton, silencing of 13-LOX type II genes, *GhLOX12* or *GhLOX13* did not result in obvious growth differences, however, when subjected to elevated concentrations of salt, they were ultra-sensitive to such stress (Shaban et al., 2018). In peanut (*Arachis hypogaea*), a 13-LOX type II, *AhLOX29* was associated with drought tolerance (Mou et al., 2022). And in maize (*Zea mays*), a 13-LOX mutant (*Zmlox10*), showed a JA independent effect on beneficial symbiont-induced systemic signaling, and this was attributed to 13-LOX-derived metabolites (Wang et al., 2020). Thus, it is reasonable to imagine a scenario in which 13-NAE-HOD and/or 13-HOD may be linked to abiotic stress responses or biotic interactions. While such hypotheses are beyond the scope of the present study, we believe that future investigations are warranted due to the possible biological implications that these metabolites might have.

In our experiments, endogenous FFA oxylipin, 9-HOD accumulated in ways that appeared to be independent of FAAH activity as might have otherwise been expected for a FAAH-derived metabolite. However, it is possible that 9-HOD might be metabolized rapidly to other products by various enzymatic steps. For example, in Arabidopsis, the hydroperoxides of linoleic acid (18:2) can be processed by allene oxide synthase, epoxy alcohol synthase, or hydroperoxide lyases, among others (Mosblech et al., 2010). Future analysis of additional metabolites derived from these routes may provide insights into the potential interaction of 9-HOD with other oxylipin pathways.

Quantification of *GhLOX* transcripts showed that *GhLOX21* transcript (but not *GhLOX10*) was elevated in *FAAH* (*Ib*- and/or *IIb*) silenced seedlings (Supplemental Figure 12B). The mis-regulation of *GhLOX21* expression upon *FAAH* suppression may further disrupt the balance of NAE18:2 and 9-NAE-HOD levels compared with those observed under normal growth conditions. Curiously, the reverse was not true; neither *GhFAAH Ib* nor *GhFAAH IIb* transcript abundances appeared to be altered by suppression of *GhLOX21.* The concept of increased *LOX* expression under *FAAH*-suppressed conditions has been reported elsewhere (Starowicz et al., 2013). In mammals, inhibition of FAAH led to reorganization of the endocannabinoid signaling machinery. Indeed, rats injected with increasing doses of URB597 (a known inhibitor of FAAH) had elevated transcript and protein levels of the 15-lipoxygenase (15-LOX), specifically at the site of injection. Overexpression of this LOX enzyme coincided with elevated levels of 15-hydroperoxy-NAE20:4 (a 15-LOX-product of anandamide) (Starowicz et al., 2013). In our work here, multiple experiments revealed that silencing of *FAAH* groups (*I* and/or *II*) or *GhFAAH* isoforms (*Ib* and/or *IIb*) could lead to higher levels of *GhLOX21* transcript, as well as elevated levels of endogenous 9-NAE-HOD. An altered homeostasis in lipid-mediator levels was clearly manifested in altered seedling growth, but how homeostasis is maintained through the coordinate expression and action of these specific FAAHs and LOX remains an area for future research.

Although the contents of several non-oxygenated 18C NAEs (e.g. NAE18:2) were the highest in seedlings where both *FAAH* groups were compromised, this did not coincide with additive growth reductions in *FAAH* (*I* and/or *II*) and FAAH (*Ib* and/or *IIb*) silenced seedlings. Instead, growth was reduced similarly in *FAAH I* and *FAAH II* silenced plants (Figure 1). On the other hand, the magnitude of changes of NAE-HODs in *FAAH*-silenced seedlings was dramatically greater compared with that of non-oxygenated/unsubstituted 18C NAEs (two orders of magnitude). Further, these levels were raised to the same “non-additive” extent in *FAAH I* and/or *FAAH II* VIGS treatments, similar to the effects in growth. Follow-up data from our genetic and pharmacological experiments consistently showed that the 9-LOX-derived NAE (9-NAE-HOD) is the bioactive factor associated with growth reduction rather than the NAE18:2 parent molecule. This notion is consistent with a previous report in Arabidopsis, where the effects of 9-NAE-HOD and NAE18:2 were compared and, 9-NAE-HOD was shown to be a more potent inhibitor of seedling growth than NAE18:2 (Keereetaweep et al., 2015). Thus, it may be that the relatively minor changes in unsubstituted NAEs amounts here in our studies with *FAAH* suppressed cotton seedlings simply reflect slight changes in NAE-HOD precursor pools, and are not directly attributable to changes in growth themselves.

In conclusion, we provide multiple lines of evidence to support a connection between NAE oxidation and hydrolysis and a role in seedling growth of upland cotton. In this case, we identified specific isoforms in broader gene families that participate in this process, and suggest that with expanded *FAAH* and *LOX* isoforms, this mechanism of growth modulation may be a general phenomenon in angiosperms. It is possible that the interaction of FAAH and LOX pathways may form additional lipid mediators that influence other physiological processes in plants as well. Certainly, the NAE signaling pathway has been shown to play a wide range of lipid mediator functions outside of the plant kingdom, including but not restricted to, longevity in roundworm (*Caenorhabditis elegans*) (Lucanic et al., 2011), satiety in mammals (Gaetani et al., 2003) and neurological and cognitive functions in vertebrates in general (Kathuria et al., 2003; Clapper et al., 2010; Servadio et al., 2016). Overall, our results here have uncovered NAE metabolic targets for crop improvement strategies aimed at seedling vigor.

## Materials and methods

### Sequence retrieval of *FAAHs* and *9-LOXes*

The upland cotton (*Gossypium hirsutum* L. cv Coker 312) *FAAH* genes used in this study have been reported previously (Arias-Gaguancela et al., 2022). Group I *FAAH* comprises *GhFAAH Ia*, *Ib*, and *Ic,* whereas Group II *FAAH* contains *GhFAAH IIa*, *IIb*, and *IIc*. Genome-wide characterization of cotton *9-LOX* genes was reported elsewhere (Shaban et al., 2018). To keep consistency, the same gene identifiers were maintained in this study. Phylogenetic analysis of the *9-LOX* coding sequences was conducted using the Phylogeny.fr online server with default settings (Dereeper et al., 2008).

### Plant material

Ten-day-old upland cotton seedlings were used for initiating all VIGS experiments. Seedlings were germinated in paper rolls immersed in water for 7 days, then those at a similar size/stage of development were moved to soil for additional seedling establishment for 3 days. Plants grew at 28°C with 16-h light/8-h dark cycles. After VIGS inoculations, they were kept overnight in a dark room at room temperature, and then transferred to a growth chamber set to 22°C, as recommended elsewhere (Gao et al., 2011; Gao & Shan, 2013). Also, previously characterized transgenic (*AtFAAH*) cotton lines, namely, AtFAAH-OE2 and AtFAAH-OE3 (Arias-Gaguancela et al., 2022) were used in this study. These seedlings germinated under the same conditions described before (Arias-Gaguancela et al., 2022) and upon VIGS-treatment they grew under the conditions described above.

### Virus-induced gene silencing of cotton *FAAHs* and *LOXes*

VIGS was used to silence multiple or single cotton *FAAH* and/or *9-LOX* genes, using the tobacco rattle virus (pYL156: TRV-RNA2) expression vector (TAIR accession: 5019327236) following the general method laid out in (Gao et al., 2011; Gao & Shan, 2013).

To silence groups of *FAAHs* and pairs of putative *9-LOX* genes, the coding sequences (CDS) of the most conserved regions (∼300 base pairs) of *FAAH I* (*GhFAAH Ia*, *Ib*, and *Ic*), *FAAH II* (*GhFAAH IIa*, *IIb*, and *IIc*), and *9LOXc1* (*GhLOX1*, *GhLOX11*), *9LOXc2* (*GhLOX10*, *GhLOX21*), or *9LOXc3* (*GhLOX8*, *GhLOX19*) groups were determined and analyzed with Sol Genomics Network VIGS online tool using the default settings (Fernandez-Pozo et al., 2015). These nucleotide regions were amplified with specific primers (Supplemental Table 1), and cloned into pYL156: TRV-RNA2 using EcoRI and BamHI restriction enzymes, resulting in VIGS constructs TRV: FAAH I, TRV: FAAH II, TRV: 9LOXc1, TRV: 9LOXc2, and TRV: 9LOXc3. Then, these vectors were electroporated in *Agrobacterium tumefaciens* GV3101 strain, and agro-delivered into cotyledons of cotton seedlings as described elsewhere (Gao et al., 2011). Briefly, *A. tumefaciens* harboring VIGS vectors were inoculated and grown in Luria broth media (Sigma-Aldrich) with antibiotics (50 µg/ml Kanamycin, 50 µg/ml Rifampicin, and 25 µg/ml Gentamycin) overnight in a shaker incubator set at 28°C, 200 RPM. Cultures were centrifuged at 13,000 RPM for 5 min, and the pellet was re-suspended in infiltration media (10 mM MES; 10 mM MgCl_2_; 100 μM acetosyringone). OD_600_ was normalized to 0.5–1. Different TRV: FAAH and/or TRV: LOX vectors were mixed in equal volumes for co-silencing experiments. The final volume of the co-silencing mixture was mixed with pYL192: TRV1 (helper vector, carries RNA polymerase for infection) in a 1:1 ratio. Then, the mixture was delivered with a needless syringe onto the abaxial side of 10-day-old cotyledons, until saturation was reached (Miao et al., 2019). All experiments included two negative controls: mock (infiltration media), and TRV: Empty (VIGS empty vector without a silencing sequence). To assess silencing timing, one VIGS vector that targets *Magnesium chelatase subunit H* (TRV: MgChIH) was used as positive control due to its visible marker (albino phenotype), as reported elsewhere (McGarry et al., 2016). Recently inoculated seedlings were kept in a dark room at room temperature overnight. Plants were transferred to a growth chamber set to 22°C with 16-h light/8-h dark cycles. Phenotypic changes were recorded at 20 days post infiltration (dpi) with a Nikon D3100 digital camera.

To identify specific *FAAH* or *9-LOX* isoforms, the 3′- or 5′- untranslated (UTRs) regions (∼100 base pairs) of individual members of *FAAH I* (*GhFAAH Ia*, *Ib*, or *Ic*), *FAAH II* (*GhFAAH IIa*, *IIb*, or *IIc*), or *9LOXc2* (*GhLOX10*, or *GhLOX21*) were used for VIGS construct design. These regions were amplified with specific primers (Supplemental Table 1), and cloned into pYL156: TRV2 using EcoRI and BamHI restriction enzymes, resulting in VIGS vectors, namely, TRV: FAAH Ia, TRV: FAAH Ib, TRV: FAAH Ic, TRV: FAAH IIa, TRV: FAAH IIb, TRV: FAAH IIc, TRV: LOX10, and TRV: LOX21. These vectors were electroporated in *A. tumefactions* GV3101 for transfection using the same steps described above. A selected TRV: FAAH and/or TRV: LOX vector was mixed with pYL192: TRV1in a 1:1 ratio, and then delivered onto the abaxial side of 10-day-old cotyledons. The same positive and negative controls described above were used for these experiments. At 20 dpi, plant phenotypes were recorded, and tissues were collected for further analysis.

### RNA extraction, cDNA production, and reverse transcription quantitative PCR

Three biological replicates (three true leaves from three different seedlings) were utilized for RNA extraction, cDNA synthesis, and RT-qPCR analysis. A modified hot-borate extraction method was used for RNA extraction as described previously (Wan & Wilkins, 1994; Arias-Gaguancela et al., 2022). Then, 100 nanograms of RNA were used for cDNA synthesis using the Applied Biosystems High Capacity cDNA Reverse Transcription Thermo Fisher kit. The resulting cDNA was normalized to 250 ng/µl and used for RT-qPCR analysis. Applied Biosystems PowerUp™ SYBR™ Green Master Mix-Fisher Scientific kit was used for the experiments following the instructions of the manufacturer. RT-qPCR amplification was conducted in Quant Studio^TM^ three system as follows; 50°C for 2 min, 95°C for 10 min, 40 cycles of 95°C for 15 s, and 60°C for 1 min. Specific primers (Supplemental Table 1) were used to quantify *FAAH* and/or *LOX* transcripts. *Ubiquitin 1* (*UBQ1*) served as the “housekeeping” gene of normalization. The delta-delta-Ct (ddCt) method was used to calculate transcript abundance (Rao et al., 2013) relative to mock-infected negative controls.

### Extraction and quantification of unsubstituted/non-oxygenated NAEs

Total lipid extraction and NAE quantification in cotton tissues were conducted as previously described (Venables et al., 2005; Kilaru et al., 2011; Keereetaweep et al., 2013, 2015; Keereetaweep & Chapman, 2016; Arias-Gaguancela et al., 2022). Briefly, pools of three true leaves from one seedling represent one biological replicate, and three biological replicates (three leaves from three seedlings each) were used for lipid extraction. Leaves were lyophilized and 600 mg of dry weight were homogenized with a solution of preheated (70°C) isopropanol with butylated hydroxytoluene (BHT) 0.01% (w/v), followed by the addition of water and chloroform in a ratio 2:1:0.45. Then, 250 ng of internal standard d4-NAE16:0 (Cayman Chemical) was added to the mixture. Lipid two-phase partitioning was conducted, and the chloroform phase was washed and recovered for separation in normal phase (NP)-HPLC on a VP 250 × 10 mm Nucleodur 100–10 column. NAE-enriched fractions were collected between 11 and 15 min, and these fractions were evaporated under N_2_ stream and derivatized with N, O-bis(trimethylsilyl) trifluoroacetamide (BSTFA) for 30 min at 50°C. Then, BSTFA was evaporated under N_2_ and derivatized lipids were re-suspended in 20 µl of hexane for analysis on an Agilent 5975C GC/MSD system equipped with a 30 m capillary column (J&W HP-5 ms GC). The GC-MS method followed the conditions described elsewhere (Arias-Gaguancela et al., 2022). NAEs were detected and identified in full scan mode, whereas quantification was performed under the single ion monitoring (SIM) mode using the diagnostic and quantitative ions for each NAE type listed in Supplemental Table 2.

### Extraction and quantification of NAE- and FFA-oxylipins

Similar to non-oxygenated NAE analysis, combined tissues from three true leaves from one seedling represented one biological replicate. In total, three biological replicates for each treatment were used in the extractions. NAE-oxylipins (13-NAE-HOD, 9-NAE-HOD) and their corresponding FFA-oxylipins (13-HOD, 9-HOD) were extracted and quantified as described previously (Kilaru et al., 2012; Christensen et al., 2013; Keereetaweep et al., 2013, 2015; Keereetaweep & Chapman, 2016; Arias-Gaguancela et al., 2022). Briefly, lyophilized tissues (900 mg dry weight per replicate) were powdered in a bead beater in 3 ml of solvent containing hexane: isopropyl alcohol (1.5:1), and BHT 0.0025% (w/v). 250 ng of deuterated FFA oxylipin 9-HOD (d_4_-9HOD-Cayman Chemical) was added to the mixture as an internal standard. To ensure full reduction of hydroperoxides to hydroxides in the lipid extracts, 200 mg of NaBH_4_ was added to the mixture, and samples were shaken and incubated for 30 min at 4°C. Following centrifugation, the hexane-rich phase was collected and subjected to reverse phase (RP)-HPLC using an EC 250/2 Nucleosil 120-5 C18 column. Oxylipin-enriched fractions were collected between 20 and 30 min, and subjected to BSTFA derivatization as described above for non-oxygenated NAEs. Oxylipins were dissolved in 20 µl of hexane, and an aliquot was subjected to GC-MS separation (Agilent 5975C GC/MSD system with capillary column J&W HP-5 ms GC as above). Full scan and SIM modes were used for the identification and quantification of oxylipins as above (Supplemental Table 2) and as described elsewhere (Kilaru et al., 2011; Keereetaweep et al., 2013, 2015; Arias-Gaguancela et al., 2022).

### Exogenous applications of NAE- and FFA- oxylipins

Ten-day-old upland cotton seedlings were irrigated with 10 ml of 13-NAE-HOD, 9-NAE-HOD, 9-HOD, and 13-HOD solutions at three different concentrations 10, 25, and 50 µM in 0.05% (v/v) DMSO (final concentration, prepared fresh from stock solutions) every 5 days. Given that these seedlings were not subjected to VIGS treatment, this plant material grew exclusively at 28°C, following a 16-h light/8-h dark cycle. A DMSO solvent control 0.05% (v/v) was used in all experiments for side-by-side comparisons at 18 days post-treatment (dpt). NAE-oxylipins (13-NAE-HOD, 9-NAE-HOD) were synthesized enzymatically from NAE18:2 (linoleoylethanolamide, Cayman Chemical) and purified by RP-HPLC as previously described by Arias-Gaguancela et al., 2022. FFA-oxylipins (13-HOD, 9-HOD) were purchased from Cayman Chemical.

### FAAH activity assays

Four biological replicates (four different leaves from four different *FAAH* and/or *LOX* silenced seedlings) were used to assay FAAH enzymatic activity. Plant homogenates were prepared as described previously (Kim et al., 2013; Arias-Gaguancela et al., 2022) with some modifications. Briefly, harvested samples were flash-frozen and ground to fine powder in liquid N_2_, and 200 mg of the tissue was mixed with 2 ml of homogenization buffer (10 mM KCl, 1 mM EDTA, 1 mM EGTA, 1 mM MgCl_2_, 400 mM sucrose, and 100 mM potassium phosphate pH 7.2, 0.6 mM DDM). Samples were incubated on ice for 20 min, and were vortexed every 5 min for 10 s. Then, samples were centrifuged for 40 min at 4°C and 2,400 X*g*. Supernatants were used as enzyme source for the assays. Pierce BCA Protein Assay Kit (Thermo Scientific) was used for protein quantification, following the instructions of the manufacturer. Samples were normalized to 100 µg of total protein, and they were incubated with 100 µM of 9-NAE-HOD substrate for 15 min at 30°C. Reactions were stopped with 10 mM of phenylmethylsulfonyl fluoride. Then, an aliquot of 15 µl was taken from the reaction and mixed with fluorescamine (3.6 mM) and milli-q-water. The mixture was incubated at room temperature for 5 min, and fluorescent values were quantified in Agilent BioTek Synergy H4 fluorimeter (Palmer et al., 2014; Murugayah et al., 2019; Arias-Gaguancela et al., 2022). A standard curve with ethanolamine was used for the conversion of fluorescent values to ethanolamine produced per mg of protein used, as described previously (Arias-Gaguancela et al., 2022).

### Multivariate and statistical analysis

PCA and Spearman's correlation analysis were performed in MetaboAnalyst online version 5.0 (https://www.metaboanalyst.ca/) (Xia et al., 2009) using the default settings. Statistical analysis was also done in MetaboAnalyst using either ANOVA with post-hoc Tukey's test.

## Accession numbers

Accession numbers of genes for this study are as follows: GhFAAH Ia (Gh_A13G0418), GhFAAH Ib (Gh_D12G0166), GhFAAH Ic (Gh_A12G0153), GhFAAH IIa (Gh_D10G1046), GhFAAH IIb (Gh_D04G0289), GhFAAH IIc (Gh_A10G0601), GhLOX1 (Gh_A02G0294), GhLOX11 (Gh_D02G0358), GhLOX10 (Gh_A13G0888), GhLOX21 (Gh_D13G1129), GhLOX8 (Gh_A09G2247), and GhLOX19 (Gh_D09G2080). Accessions can be retrieved from ccNET database (http://structuralbiology.cau.edu.cn/gossypium/).

## Supplemental data

The following materials are available in the online version of this article.

**Supplemental Figure S1.** Primary leaves of *9-LOX* and *FAAH* silenced cotton seedlings.

**Supplemental Figure S2.** Leaf measurements of primary leaves detached from *9-LOX* and *FAAH* silenced cotton seedlings.

**Supplemental Figure S3.** Transcripts of *9-LOX* clusters in *FAAH/LOX* co-silencing experiments.

**Supplemental Figure S4.** Transcripts of *FAAH* genes in *FAAH/LOX* co-silencing experiments.

**Supplemental Figure S5.** Total (unsubstituted/non-oxygenated) NAE content of inoculated seedlings with TRV: FAAH (I and/or II) and/or TRV: 9LOX (c1, c2, c3), or TRV: 9LOX (all clusters together).

**Supplemental Figure S6.** Profile of individual non-oxylipin NAE types in seedlings inoculated with TRV: FAAH (I and/or II) and/or TRV: 9LOX (c1, c2, c3), or TRV: 9LOX (all clusters together).

**Supplemental Figure S7.** Diagram describing NAE18:2 and 9-NAE-HOD patterns along with seedling growth phenotypes in silencing experiments with TRV: FAAH (I and/or II), and/or TRV: 9-LOX groups (c1, c2, or c3).

**Supplemental Figure S8.** Leaf measurements of primary leaves detached from *FAAH I* (*Ia*, *Ib*, or *Ic*) or *FAAH II* (*IIa*, *IIb*, or *IIc*) silenced cotton seedlings.

**Supplemental Figure S9.** Transcripts of *FAAH* genes in *FAAH I* (*Ia*, *Ib*, or *Ic*) or *FAAH II* (*IIa*, *IIb*, or *IIc*) silenced cotton seedlings.

**Supplemental Figure S10.** Total (unsubstituted/non-oxygenated) NAE content of *FAAH* (*Ia*, *Ib*, and/or *Ib*) or *FAAH* (*IIa*, *IIb*, and/or *IIb*) silenced tissues.

**Supplemental Figure S11.** Leaf measurements of primary leaves detached from *GhLOX* (*10 or 21*) and/or *GhFAAH* (*Ib* and/or *IIb*) silenced cotton seedlings.

**Supplemental Figure S12.** Transcripts of *9-LOX* or *FAAH* genes in *GhLOX* (*10* or *21*) and/or *GhFAAH* (*Ib* and/or *IIb*) silenced cotton seedlings.

**Supplemental Figure S13.** Total (unsubstituted/non-oxygenated) NAE content of *FAAH* (*Ib*, and/or *IIb*) and/or *9LOXc2* (*GhLOX10*, or *GhLOX21*) silenced tissues.

**Supplemental Figure S14.** Diagram describing NAE18:2 and 9-NAE-HOD patterns along with seedling growth phenotypes in silencing experiments with TRV: FAAH (Ib, and/or IIb) and/or TRV: LOX10, or TRV: LOX21.

**Supplemental Table S1.** List of primers used in this study.

**Supplemental Table S2.** Diagnostic/quantification ions and retention times for endogenous NAEs (unsubstituted/non-oxygenated), NAE-oxylipins and FFA-oxylipins.

## Supplementary Material

kiac556_Supplementary_Data
