# Supplementary material for: Fatty acid amide hydrolase and 9-lipoxygenase modulate cotton seedling growth by ethanolamide oxylipin levels
Source: Plant Physiol. 2022 Dec 6;191(2):1234–53. doi: 10.1093/plphys/kiac556 (PMC9922431; doi:10.1093/plphys/kiac556)
Supplement: kiac556_Supplementary_Data [file kiac556_supplementary_data.pdf]

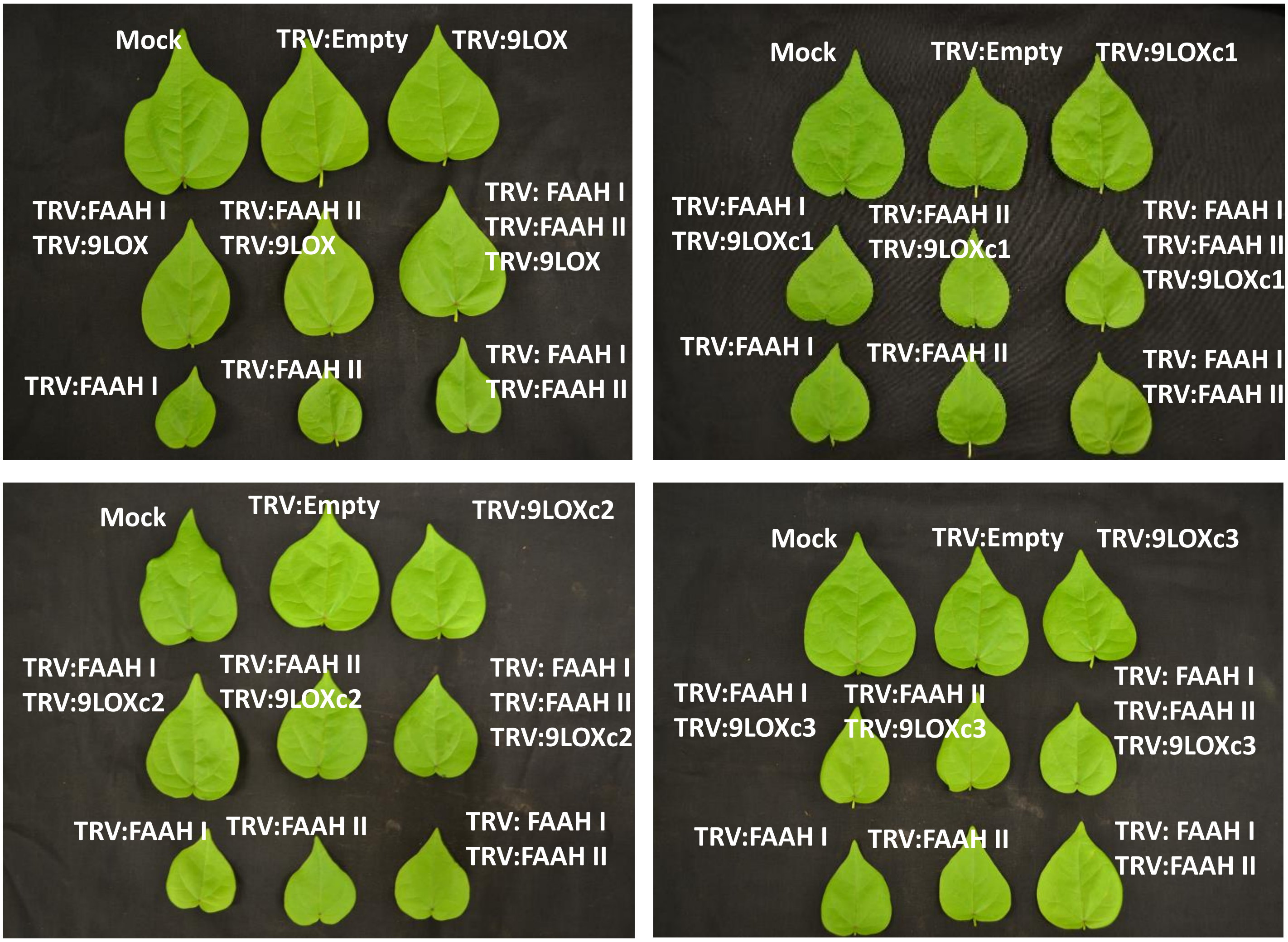

Supplemental Figure S1. Primary leaves of *9-LOX* and *FAAH* silenced cotton seedlings. Representative images of detached primary leaves from silencing experiments of TRV: 9LOXc1, TRV: 9LOXc2, or TRV: 9LOXc3, TRV: 9LOX and/or TRV: FAAH I and/or II at 20 dpi.

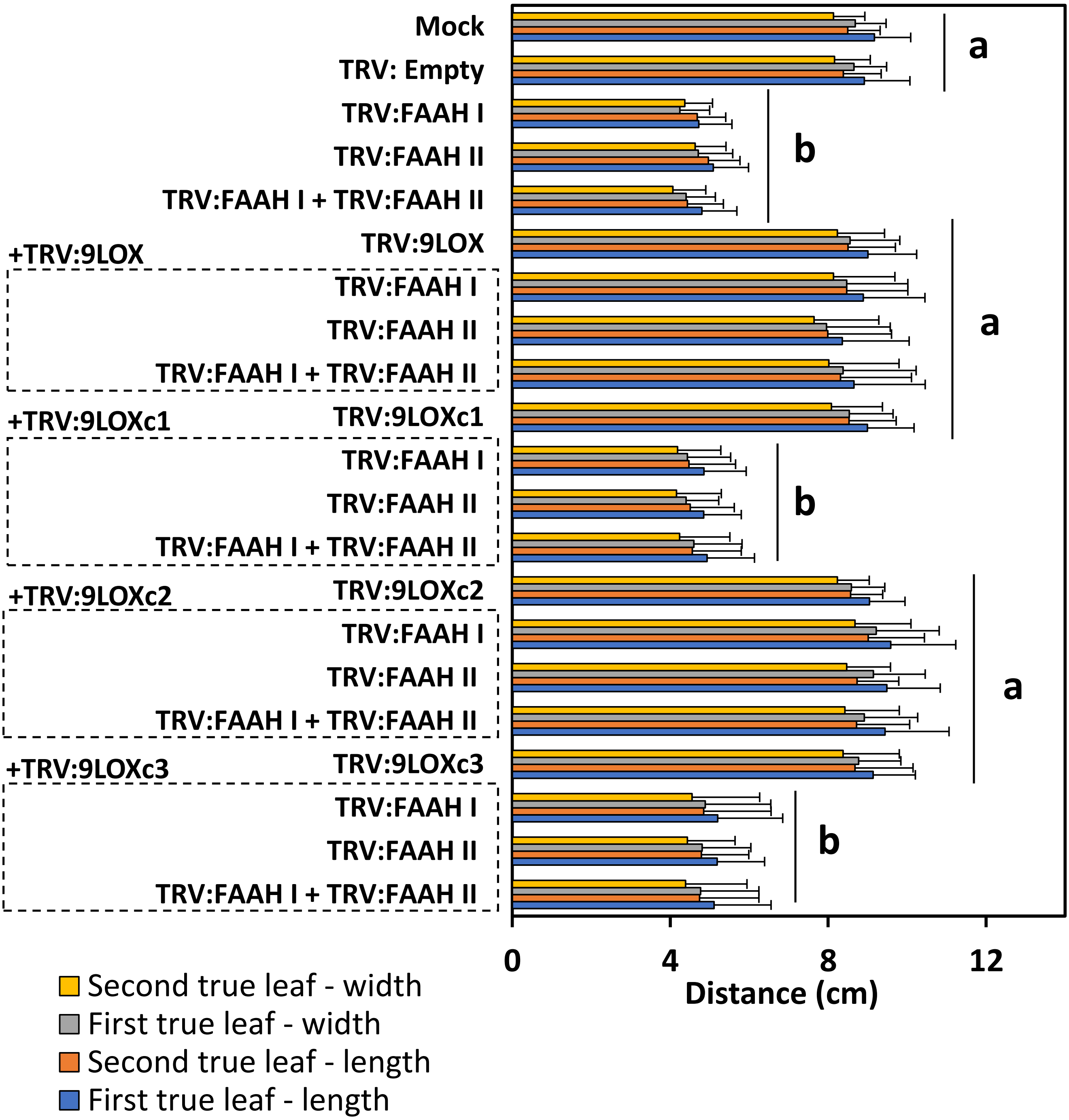

Supplemental Figure S2. Leaf measurements of primary leaves detached from *9-LOX* and *FAAH* silenced cotton seedlings. Leaf width and length (n=13) measurements in VIGS treated seedlings. Error bars represent the standard deviation (SD). Different letters denote significant differences ($P<0.05$) by ANOVA with Tukey's post-hoc test.

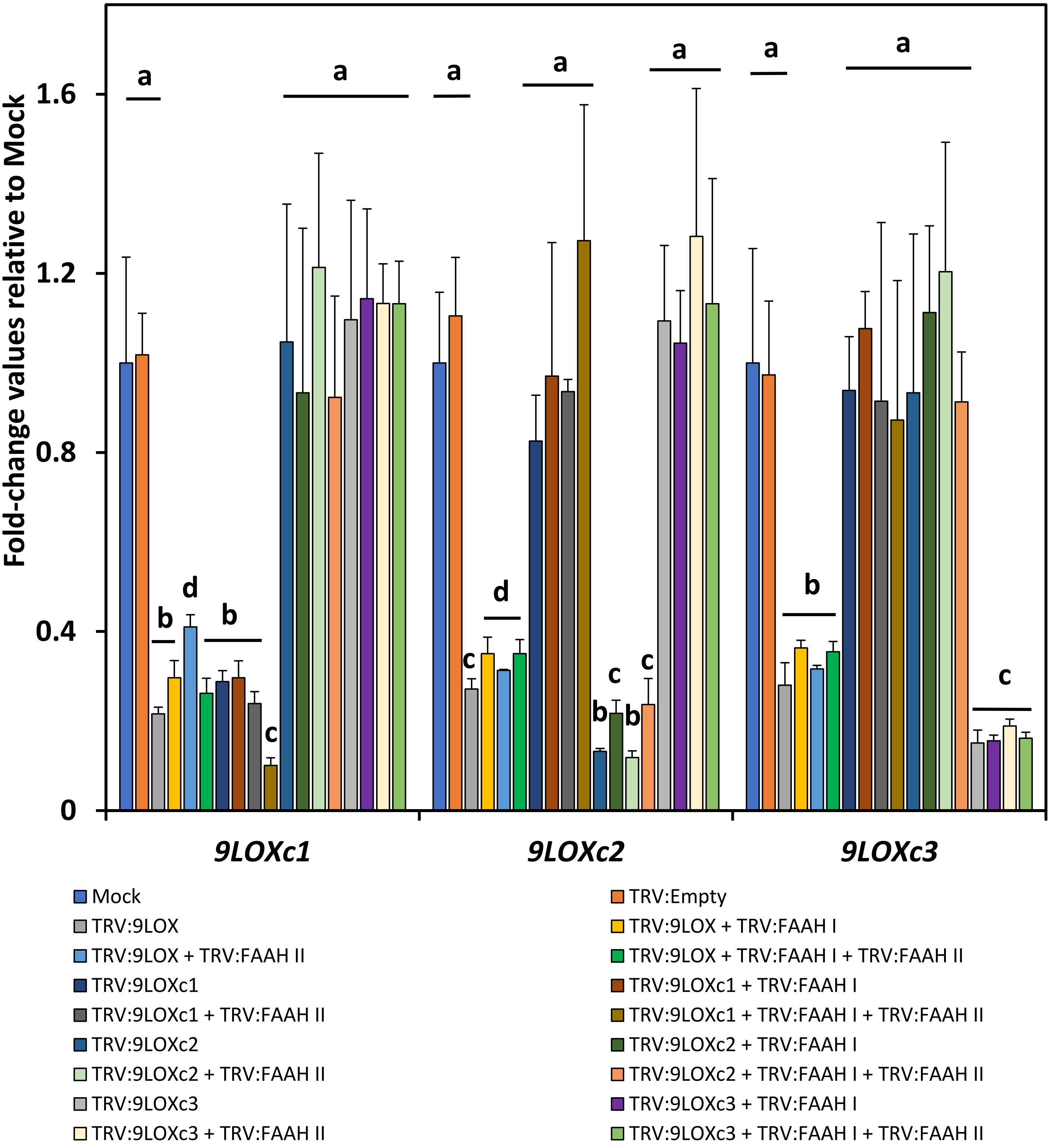

Supplemental Figure S3. Transcripts of *9-LOX* clusters in *FAAH/LOX* co-silencing experiments. RT-qPCR was used to assess *9-LOX* transcripts. *UBQ1* was used as the housekeeping gene of normalization. Calculations were made with the ddCt method, and values are presented as fold-change values relative to mock negative control. Error bars represent the standard deviation (SD). Different letters denote significant differences ($P<0.05$, n=3) by ANOVA with Tukey's post-hoc test.

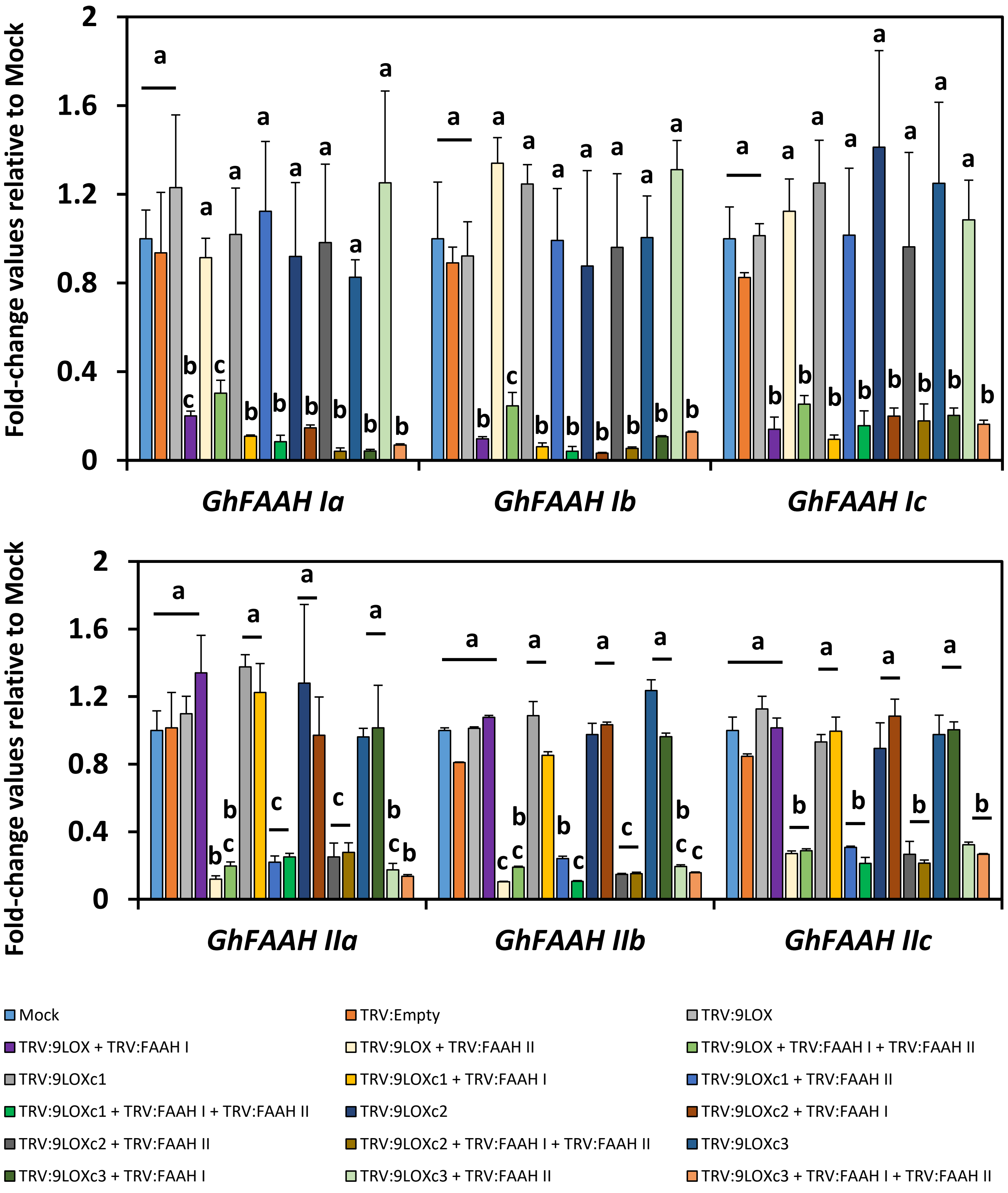

Supplemental Figure S4. Transcripts of *FAAH* genes in *FAAH/LOX* co-silencing experiments. RT-qPCR was used to assess *FAAH* transcripts. *UBQ1* was used as the housekeeping gene of normalization. Calculations were made with the ddCt method, and values are presented as fold-change values relative to mock negative control. Error bars represent the standard deviation (SD). Different letters denote significant differences ($P<0.05$, n=3) by ANOVA with Tukey's post-hoc test.

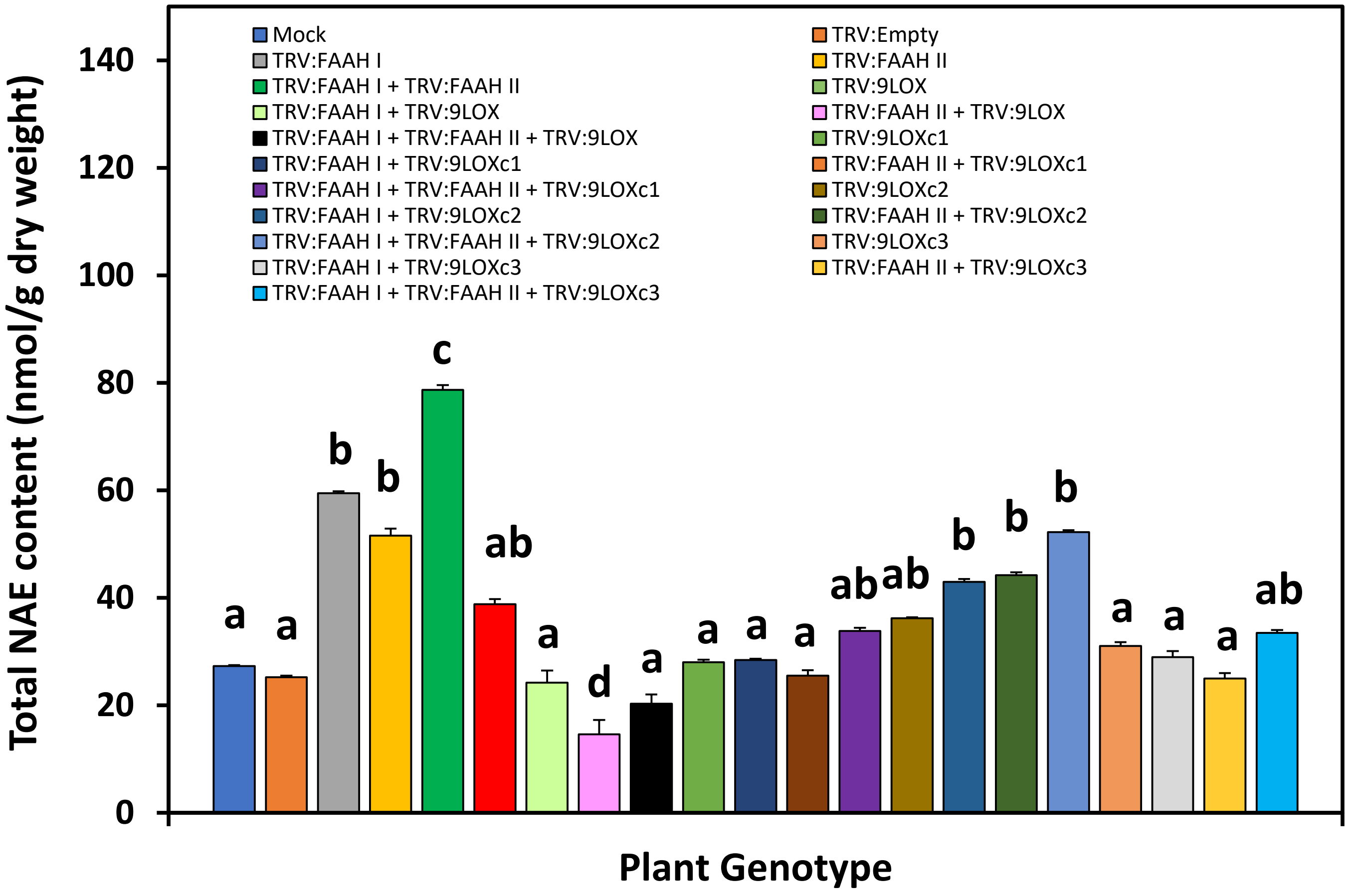

Supplemental Figure S5. Total (unsubstituted/ non-oxygenated) NAE content of inoculated seedlings with TRV: FAAH (I and/or II) and/or TRV: 9LOX (c1, c2, c3), or TRV: 9LOX (all clusters together). Error bars represent the standard deviation (SD). Different letters denote significant differences (*P*<0.05, n=3) by ANOVA with Tukey's post-hoc test.

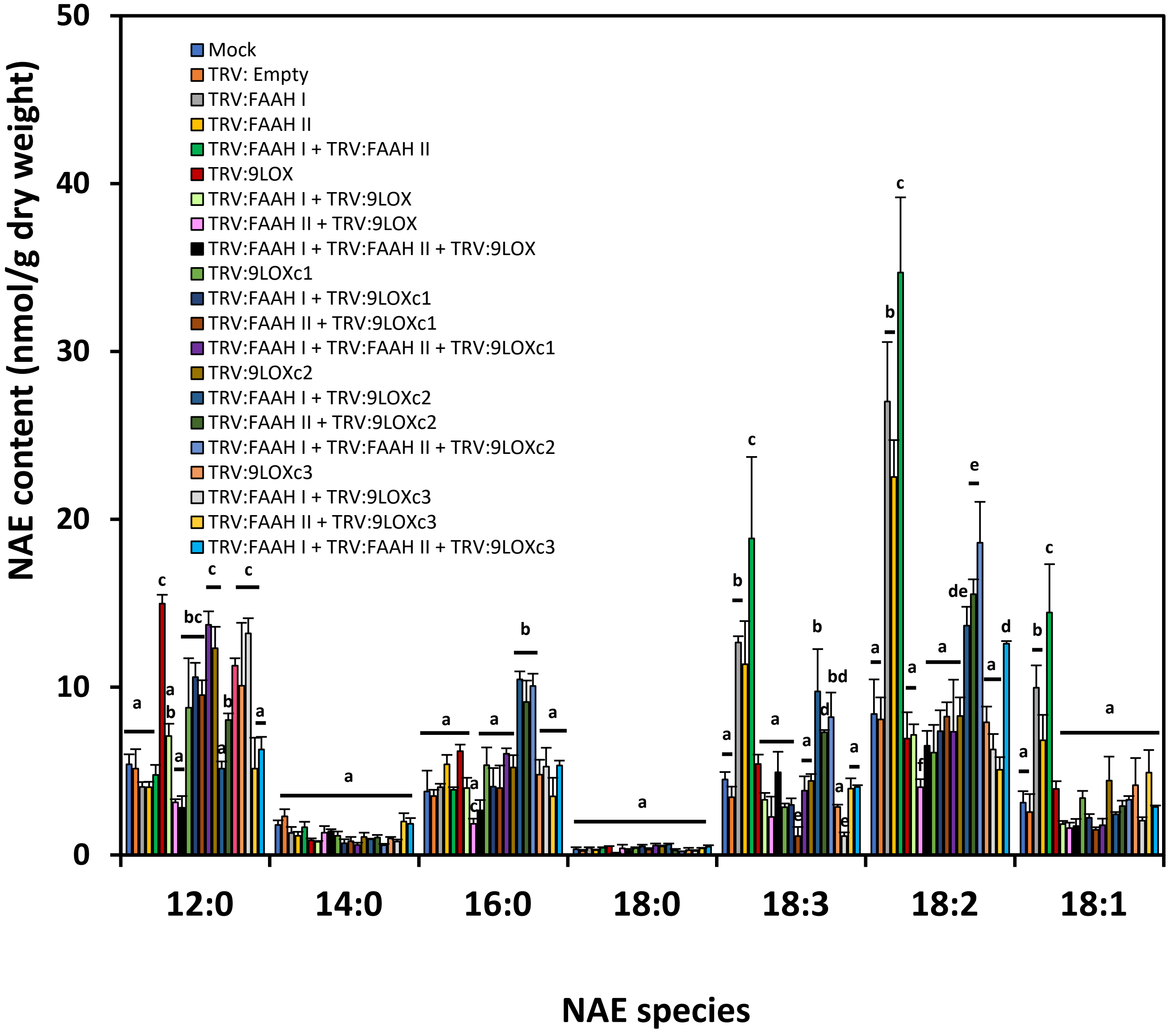

Supplemental Figure S6. Profile of individual non-oxylipin NAE types in seedlings inoculated with TRV: FAAH (I and/or II) and/or TRV: 9LOX (c1, c2, c3), or TRV: 9LOX (all clusters together). Error bars represent the standard deviation (SD). Different letters denote significant differences (P<0.05, n=3) by ANOVA with Tukey's post-hoc test.

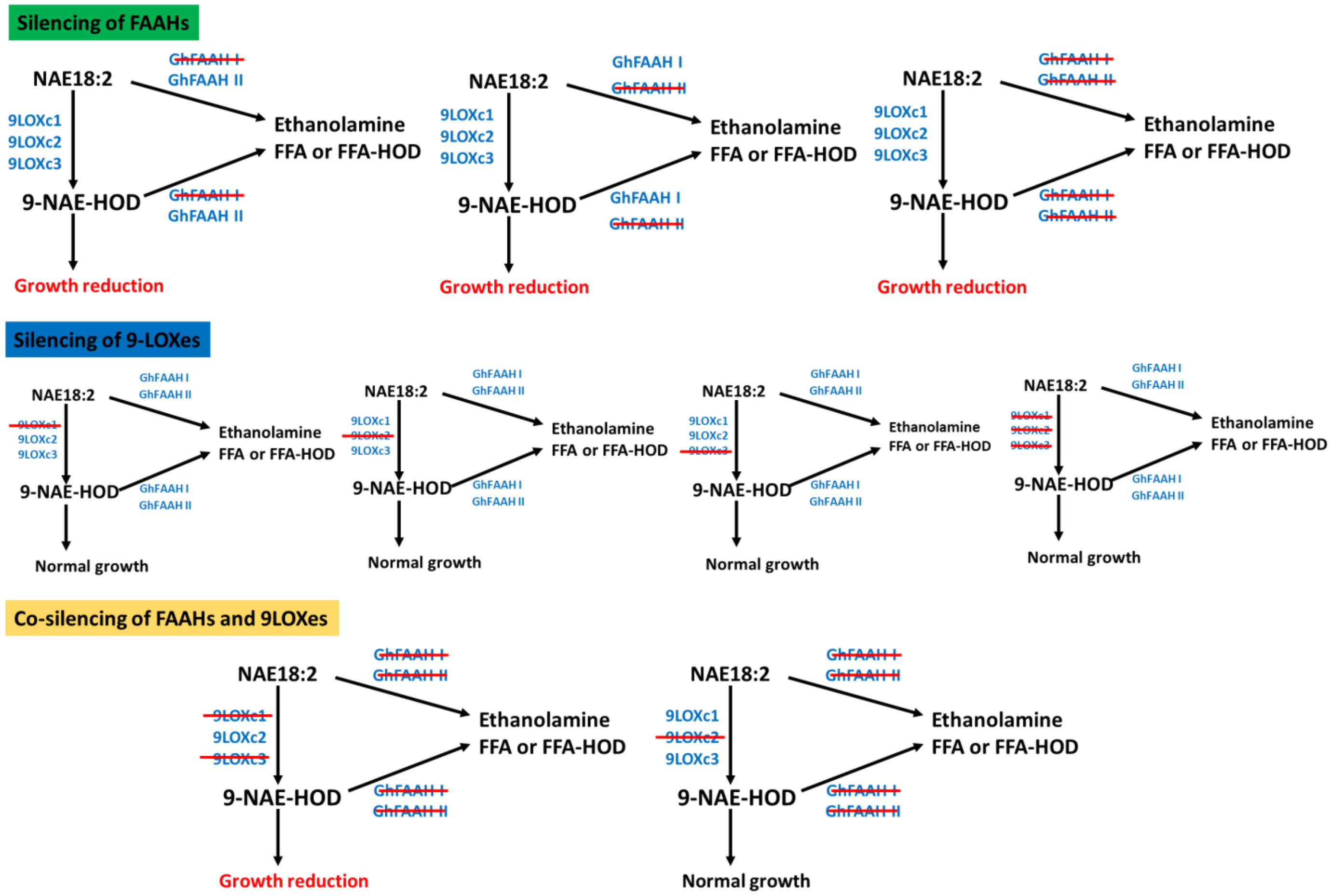

Supplemental Figure S7. Diagram describing NAE18:2 and 9-NAE-HOD patterns along with seedling growth phenotypes in silencing experiments with TRV: FAAH (I and/or II), and/or TRV: 9-LOX groups (c1, c2, or c3).

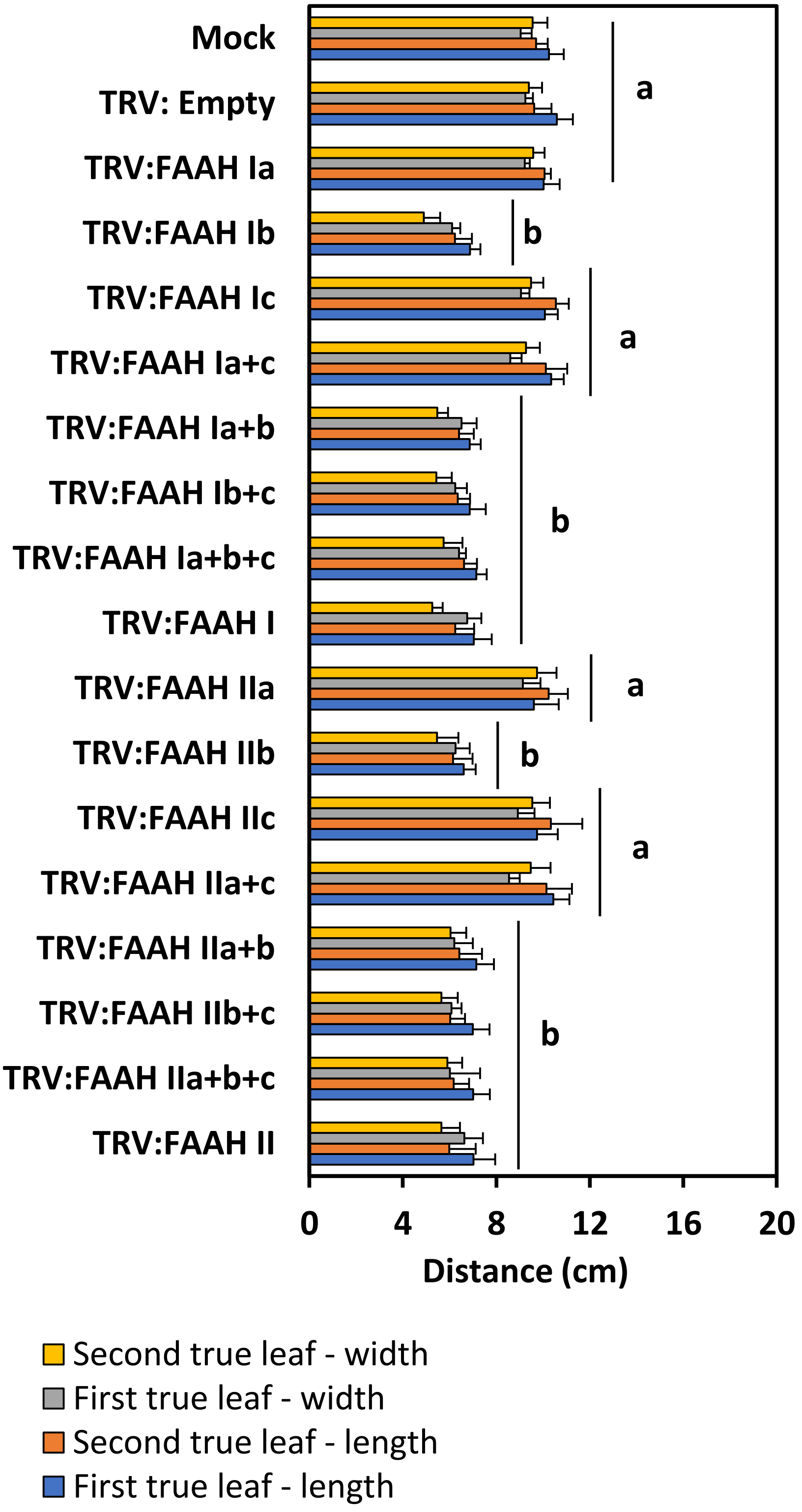

Supplemental Figure S8. Leaf measurements of primary leaves detached from *FAAH I* (*Ia*, *Ib*, or *Ic*) or *FAAH II* (*IIa*, *IIb*, or *IIc*) silenced cotton seedlings. Leaf size (n=13) measurements made for the VIGS experiments. Error bars represent the standard deviation (SD). Different letters denote significant differences ($P<0.05$) by ANOVA with Tukey's post-hoc test.

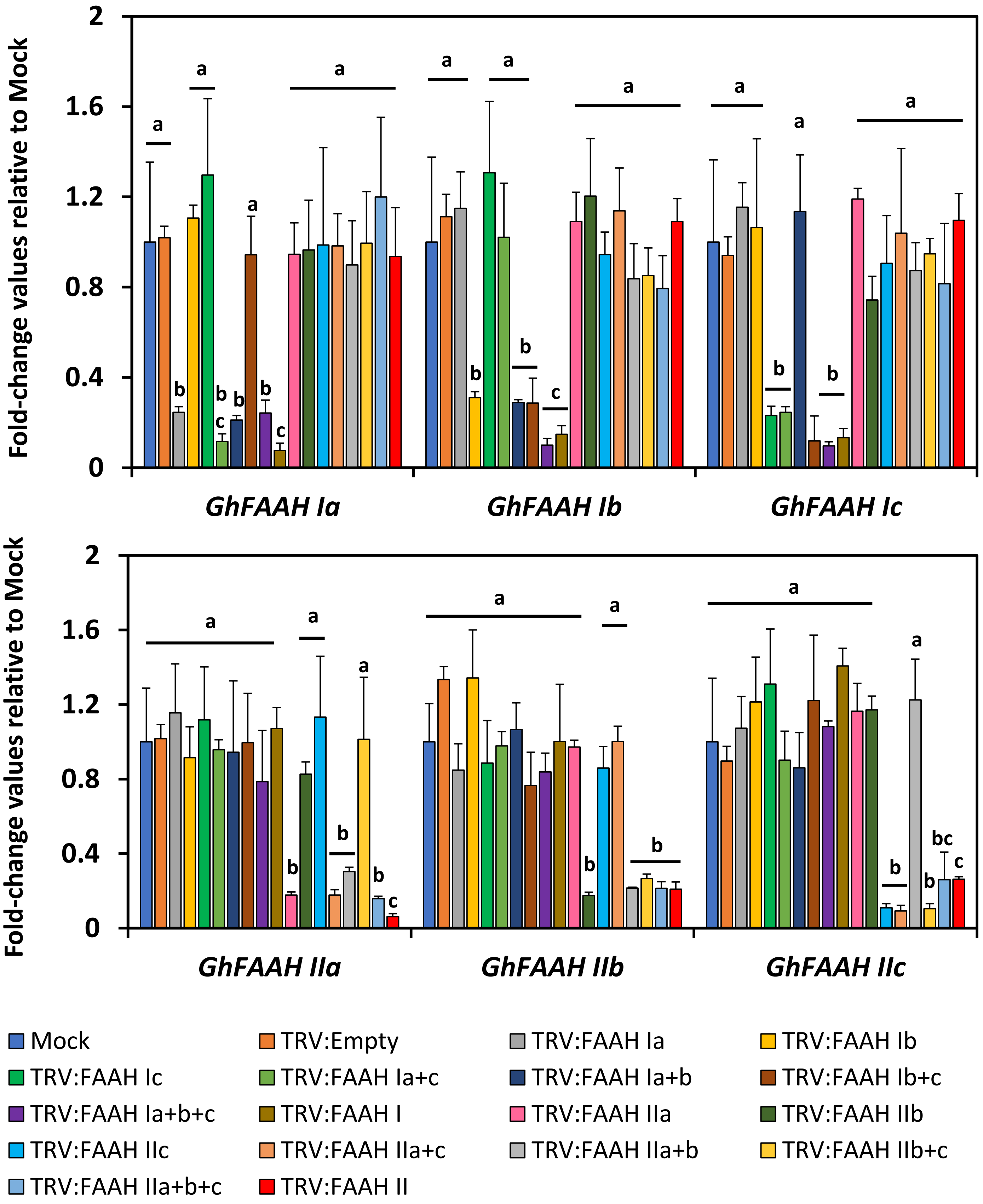

Supplemental Figure S9. Transcripts of *FAAH* genes in *FAAH I* (*Ia*, *Ib*, or *Ic*) or *FAAH II* (*IIa*, *IIb*, or *IIc*) silenced cotton seedlings. RT-qPCR was used to measure *FAAH* transcripts in all treatments. *UBQ1* was used as the housekeeping gene of normalization. Calculations were made with the ddCt method. Error bars represent the standard deviation (SD). Different letters denote significant differences ($P<0.05$, n=3) by ANOVA with Tukey's post-hoc test.

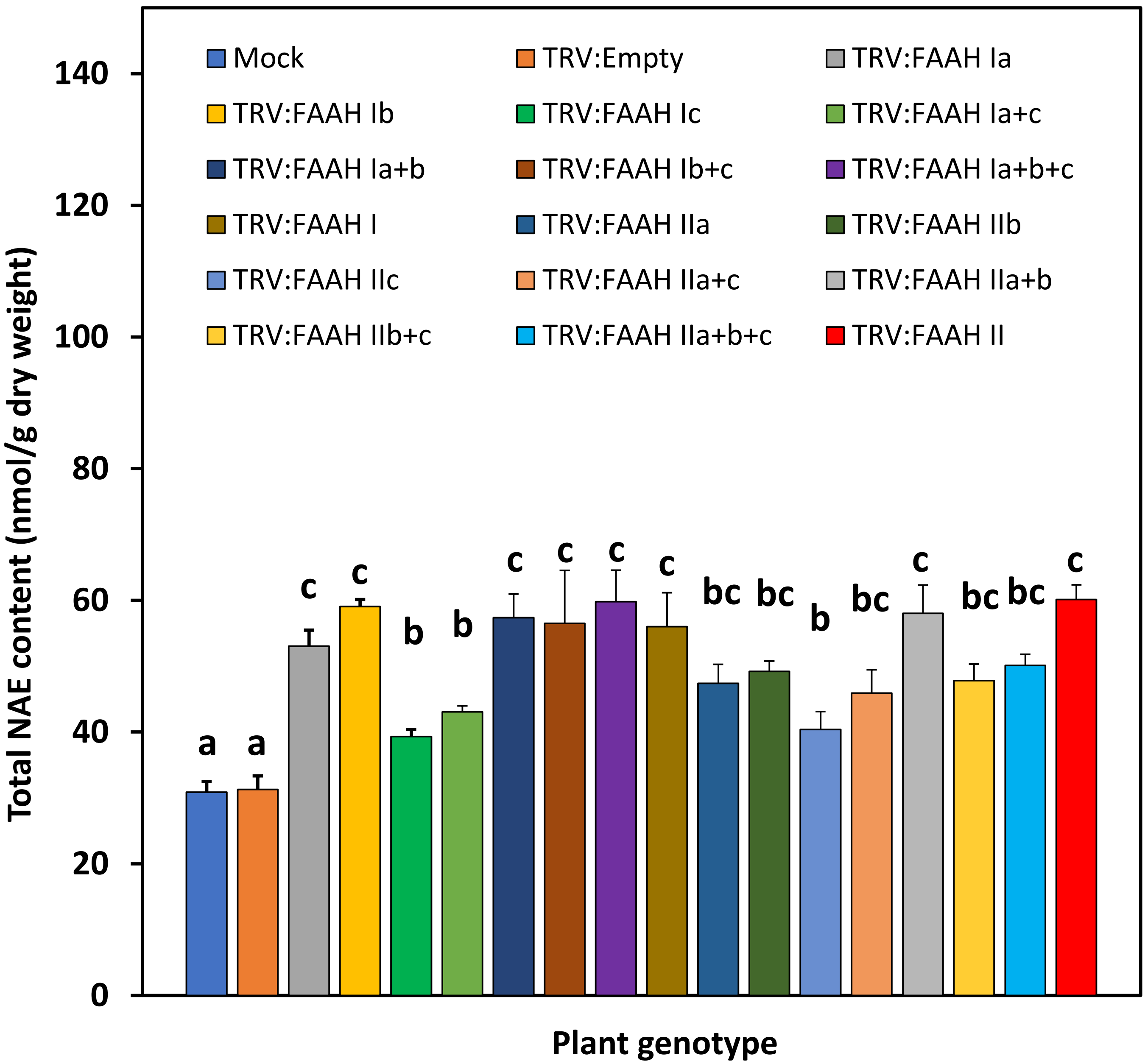

Supplemental Figure S10. Total (unsubstituted/non-oxygenated) NAE content of *FAAH* (*Ia*, *Ib*, and/or *Ib*) or *FAAH* (*IIa*, *IIb*, and/or *IIb*) silenced tissues. Error bars represent the standard deviation (SD). Different letters denote significant differences (P<0.05, n=3) by ANOVA with Tukey's post-hoc test.

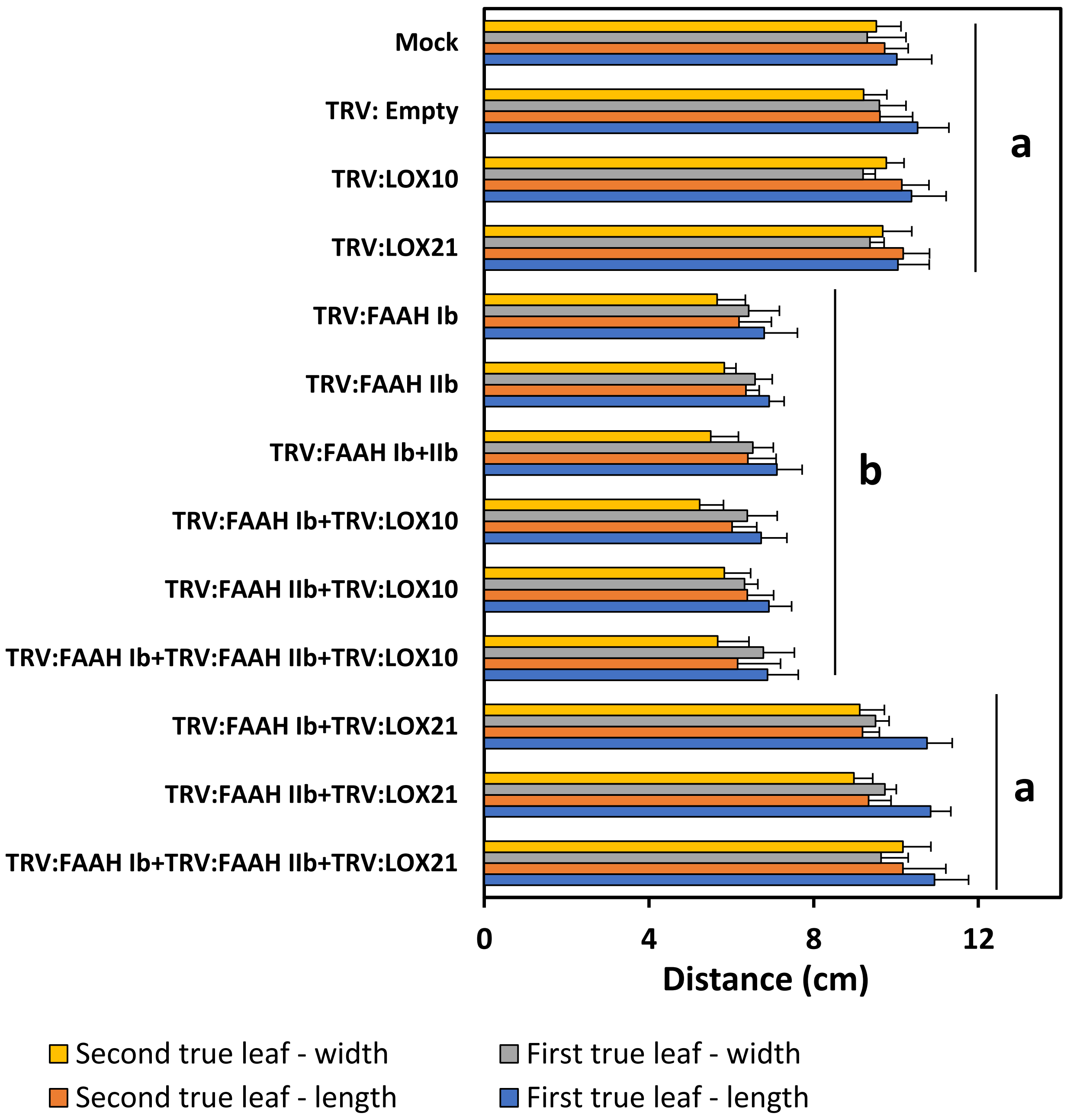

Supplemental Figure S11. Leaf measurements of primary leaves detached from *GhLOX* (*10 or 21*) and/or *GhFAAH* (*Ib* and/or *IIb*) silenced cotton seedlings. Leaf size measurements (n=13) for VIGS experiments. Error bars represent the standard deviation (SD). Different letters denote significant differences (*P*<0.05) by ANOVA with Tukey's post-hoc test.

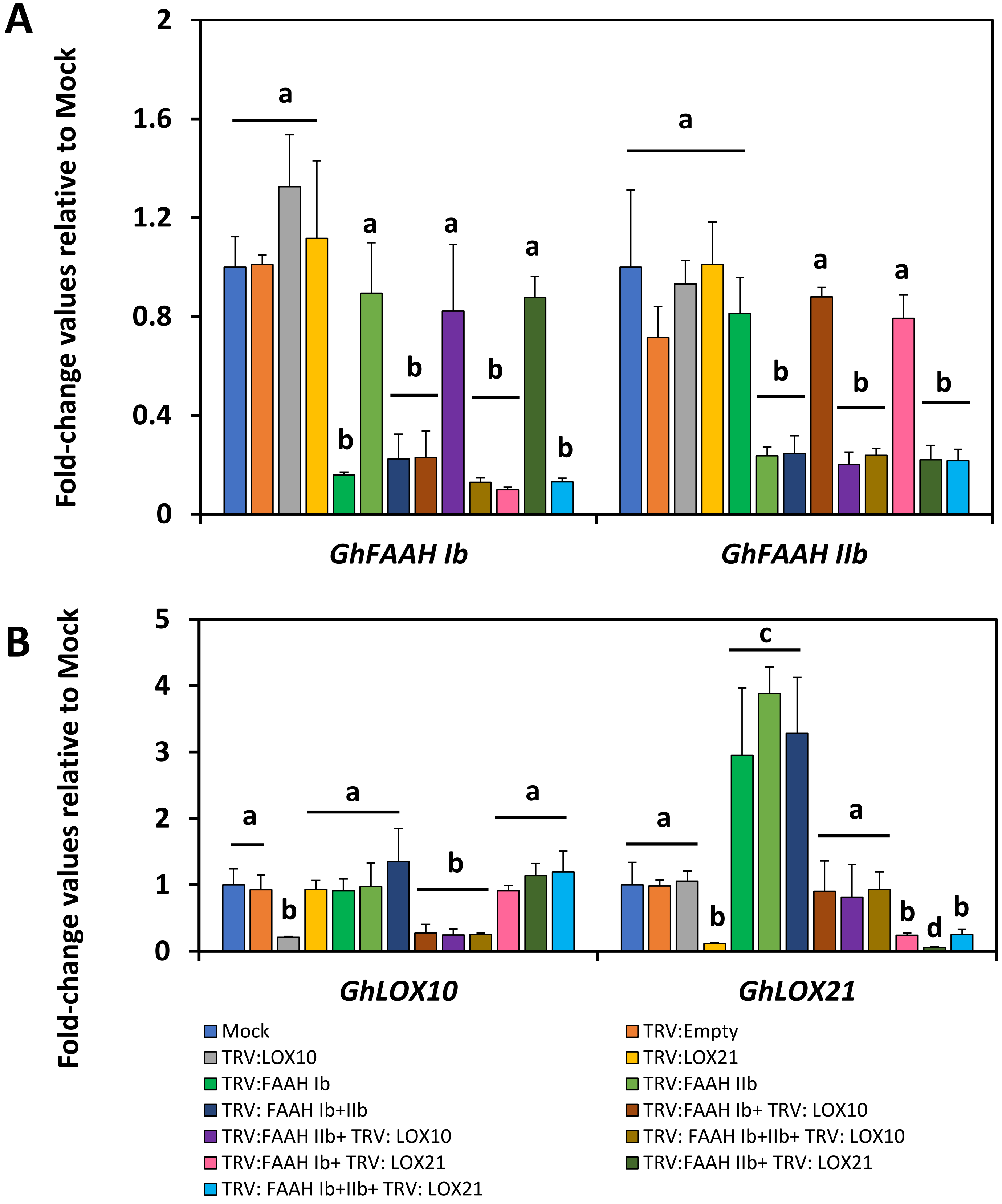

Supplemental Figure S12. Transcripts of *9-LOX* or *FAAH* genes in *GhLOX* (*10* or *21*) and/or *GhFAAH* (*Ib* and/or *IIb*) silenced cotton seedlings. A) RT-qPCR to measure *GhFAAH Ib*, *GhFAAH IIb* or B) *GhLOX10*, *GhLOX21* transcripts. *UBQ1* was used as the housekeeping gene of normalization. Calculations were made with the ddCt method. Error bars represent the standard deviation (SD). Different letters denote significant differences (P<0.05, n=3) by ANOVA with Tukey's post-hoc test.

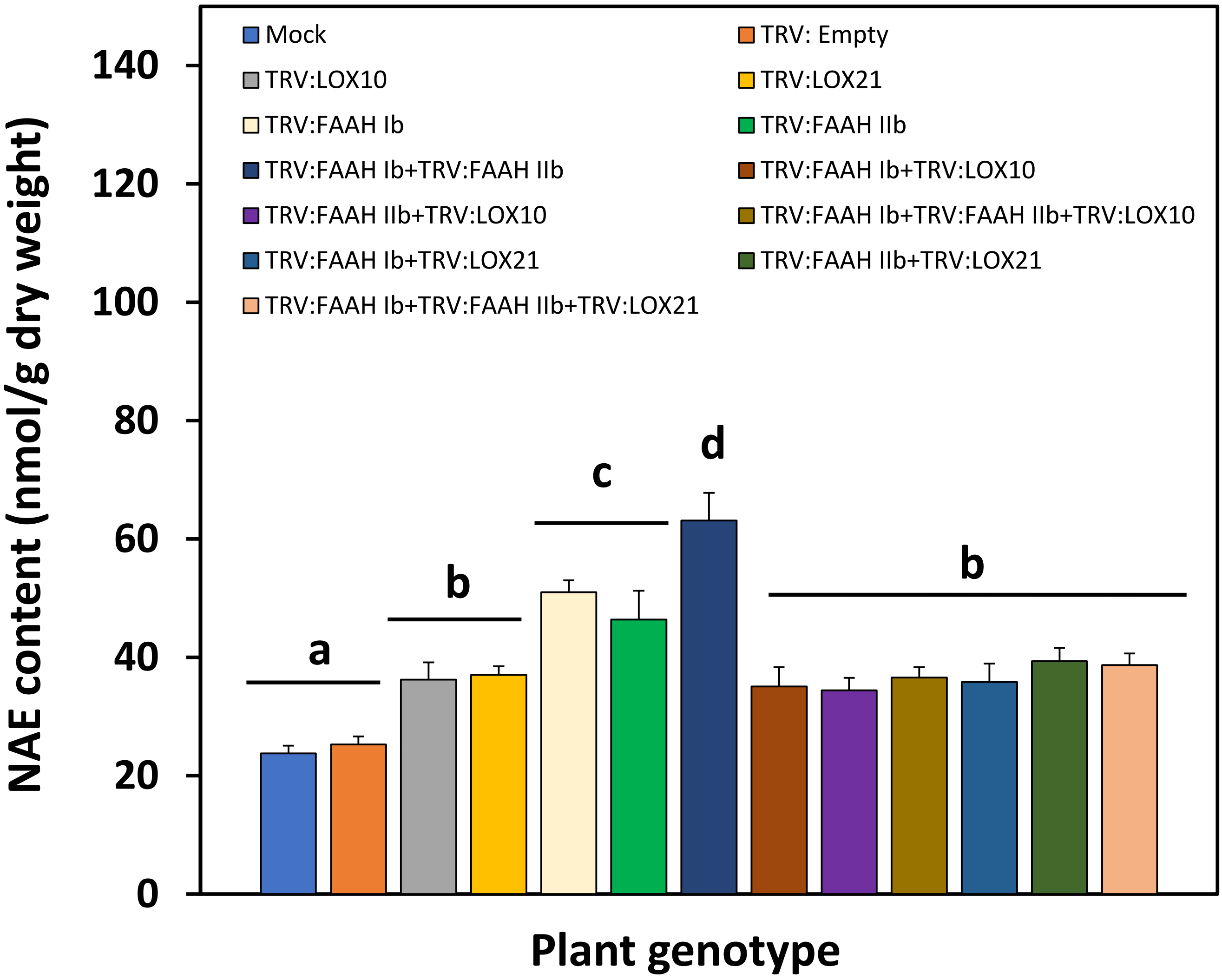

Supplemental Figure S13. Total (unsubstituted/non-oxygenated) NAE content of *FAAH* (*Ib*, and/or *IIb*) and/or *9LOXc2* (*GhLOX10*, or *GhLOX21*) silenced tissues. Error bars represent the standard deviation (SD). Different letters denote significant differences (*P*<0.05, n=3) by ANOVA with Tukey's post-hoc test.

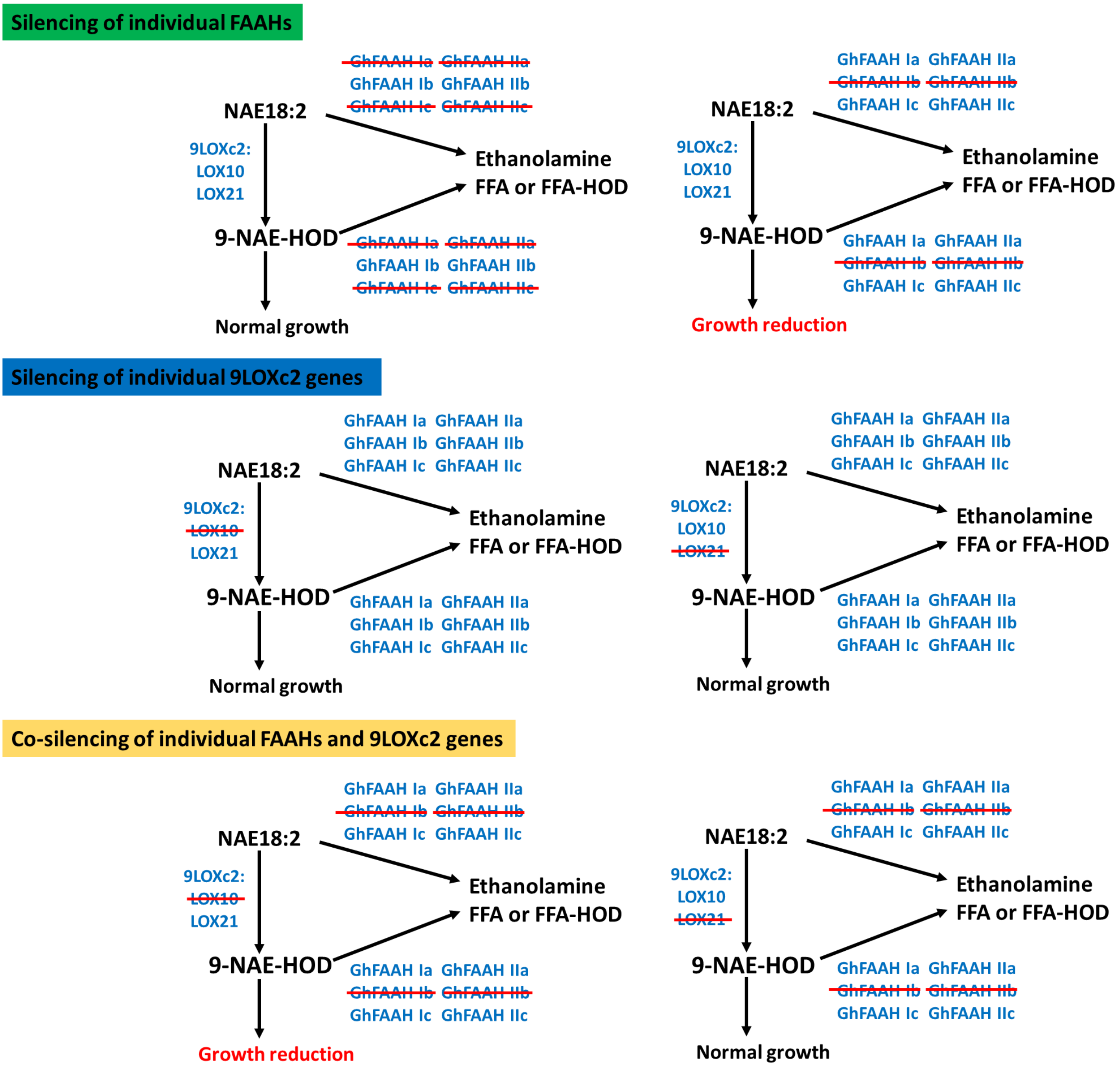

Supplemental Figure S14. Diagram describing NAE18:2 and 9-NAE-HOD patterns along with seedling growth phenotypes in silencing experiments with TRV: FAAH (Ib, and/or IIb) and/or TRV: LOX10, or TRV: LOX21.

Supplemental Table S1. List of primers used in this study.

List of primers		
**Primer name**	**Sequence**	**Purpose/Reference**
VIGS_FAAH-I_FWD	ttaccGAATTCAGGACAAACATGGGAGGA	Vector construction of TRV:
VIGS_FAAH-I_REV	catggGGATCCTCCATGGCTTTTTGACAGAA	FAAH I/ this study
VIGS_FAAH-II_FWD	ttaccGAATTCCGCATGGCTGGATTTCTTGT	Vector construction of TRV:
VIGS_FAAH-II_FWD	catggGGATCCGCGTCGGAAGCTGCTGGAAT	FAAH II/ this study
F_VIGS_GhFAAH-Ia	ttaccGAATTCTGCCAAAGACCTAAAATGTCA	Vector construction of TRV:
R_VIGS_GhFAAH-Ia	catggGGATCCAAAAGAAACATGGGCAATGG	FAAH Ia/ this study
fwd_VIGS_GhFAAH-Ib	ttaccGAATTCTGCTATCATTTCGACAAACCA	Vector construction of TRV:
rev_VIGS_GhFAAH-Ib	catggGGATCCTGGTAAAACTGAAGTCAATAACAG	FAAH Ib/ this study
fwd_VIGS_GhFAAH-Ic	ttaccGAATTCTCGATTTGGACCACAAAGTATG	Vector construction of TRV:
rev_VIGS_GhFAAH-Ic	catggGGATCCCTCTGATAAAGGAAGTGGTTGG	FAAH Ic/ this study
F_VIGS_GhFAAH-IIa	ttaccGAGTTTAATAGAAACTTTTA	Vector construction of TRV:
rev_VIGS_GhFAAH-IIa	catggTTAATGTCTAAGGCCTCAA	FAAH IIa/ this study
fwd_VIGS_GhFAAH-IIb	ttaccGAATTCCAGGGTTCATGTGCTACTTGC	Vector construction of TRV:
rev_VIGS_GhFAAH-IIb	catggGGATCCTTAATGTCTAAGGCCTCAATTACA	FAAH IIb/ this study
F_VIGS_GhFAAH-IIc	ttaccGAATTCAGTGGTTGAACTGTATTGACTTTGA	Vector construction of TRV:
R_VIGS_GhFAAH-IIc	catggGGATCCATTGCTGCAAGGACCAAGAT	FAAH IIc/ this study
F_LOX_GhLOX10_VIGS	ttaccGAATTCCTCAATCTAATGCATTTATCTTCAAA	Vector construction of TRV:
R_LOX_GhLOX10_VIGS	catggGGATCCCATATGACATCGCCTGATCC	LOX10/ this study
F_LOX_GhLOX21_VIGS	ttaccGAATTCTCAATCTGATGCATTTATCTTCAA	Vector construction of TRV:
R_LOX_GhLOX21_VIGS	catggGGATCCTGTTGCACTAATAGCAAACTTTTATTT	LOX21/ this study
FWD_LOX_C1	ttaccGAATTCTGGTGGCCCGAGATGAAGACA	Vector construction of TRV:
REV_LOX_C1	catggGGATCCGAGGTATATTTCATCGGTTGTG	9LOXc1/ this study
FWD_LOX_C2	ttaccGAATTCCCAAGTCAGATCCCAAGACTGA	Vector construction of TRV:
REV_LOX_C2	catggGGATCCGGAAAATTTCTTTGAGCATCTCC	9LOXc2/ this study
FWD_LOX_C3	ttaccGAATTCAGGGGTCATGATTTTCATCGT	Vector construction of TRV:
REV_LOX_C3	catggGGATCCTTCATAAAGATCAAGTACATC	9LOXc3/ this study
fwd_EF_VIGS_GhFAAH-Ia	TGCAGGTTACAGCCTATCTCA	RT-qPCR for GhFAAH-Ia /
rev_EF_VIGS_GhFAAH-Ia	TCAACTGCAGAAGCCAAATG	(Arias-Gaguancela et al., 2022)
fwd_EF_VIGS_GhFAAH-Ib	TCGATACGCTCGCTAAATCC	RT-qPCR for r GhFAAH-Ib /
rev_EF_VIGS_GhFAAH-Ib	GGTGCATTATCCTTCGCCTA	(Arias-Gaguancela et al., 2022)
fwd_EF_VIGS_GhFAAH-Ic	AATTCACGAGAAAATAAATCAACCA	RT-qPCR for GhFAAH-Ic /
rev_EF_VIGS_GhFAAH-Ic	TTTAACGGTCGACAATTCCAG	(Arias-Gaguancela et al., 2022)
fwd_EF_VIGS_GhFAAH-IIa	GCAGCGCAGGGTGGGAACCTC	RT-qPCR for GhFAAH-IIa /
rev_EF_VIGS_GhFAAH-IIa	AGTCAGTTGCCGGTCATTTCA	(Arias-Gaguancela et al., 2022)
fwd_EF_VIGS_GhFAAH-IIb	CCGCCACTTTATAGGTTCATGA	RT-qPCR for GhFAAH-IIb /
rev_EF_VIGS_GhFAAH-IIb	GGTGGGTGGTGATGGTTAGA	(Arias-Gaguancela et al., 2022)
fwd_EF_VIGS_GhFAAH-IIc	TCAATGATGAAATGACCGACA	RT-qPCR for GhFAAH-IIc /
rev_EF_VIGS_GhFAAH-IIc	CCGATGTGATGCACAGTTTC	(Arias-Gaguancela et al., 2022)
FWD_LOX_Gh_A02G0294_C1	CCAGCTCATCGCTACAATCA	RT-qPCR for 9-LOXc1 / this
REV_LOX_Gh_A02G0294_C1	TCCCATTCATTGAGCTTTCC	study
FWD_LOX_Gh_A13G0888_C2	TGGAAAGAACTCCGAGAGGA	RT-qPCR for 9-LOXc2 / this
REV_LOX_Gh_A13G0888_C2	GGCATGAACCGACGACTTAT	study
FWD_LOX_Gh_D09G2080_C3	ACCAGGCACCAAAGAATACG	RT-qPCR for 9-LOXc3 / this
REV_LOX_Gh_D09G2080_C3	TTCCCGAATCTTTCAAATGC	study
FWD_LOX_GhLOX10_qpcr1	CCCCAACCCCTTTTTATCTC	RT-qPCR for GhLOX10 / this
REV_LOX_GhLOX10_qpcr1	AGCAAATGGCTCAGCATCTC	study
FWD_LOX_GhLOX21_qpcr1	AGTTTGCCACATCCGAACG	RT-qPCR for GhLOX21 / this
REV_LOX_GhLOX21_qpcr1	CCAATGGCTTATGAGTTGATGA	study
FWD_UBQ1	TGGAGAGCTCGGATACGATT	RT-qPCR - Housekeeping gene /
REV_UBQ1	CACCACGAAGACGAAGAACA	(Arias-Gaguancela et al., 2022)

Supplemental Table S2. Diagnostic/quantification ions and retention times for endogenous NAEs (unsubstituted/non-oxygenated), NAE-oxylipins and FFA-oxylipins.

Lipid molecule	Qion $[M - CH3]^+$	$[M]^+$	Other diagnostic ions		Retention time (min)
NAE18:3	378	393	303	-	16.108
NAE18:2	380	395	305	-	16.067
NAE18:1	382	397	307	-	16.006
NAE18:0	384	399	309	-	16.334
NAE16:0	356	371	328	281	13.951
NAE14:0	328	343	300	253	11.580
NAE12:0	300	315	225	272	9.607
NAE16:0 d4	360	375	-	-	13.833
9-HOD	317	440	425	369, 253	12.456
13-HOD	369	440	425	253	12.804
9-NAE-HOD	360	483	468	116	18.634
13-NAE-HOD	412	483	468	116	18.876
9-HOD d4	229	444	429	-	12.427
